# Extracellular vesicle‐LncRNA HOTAIR modulates esophageal cancer chemoresistance and immune microenvironment via miR‐375/CDH2 pathway

**DOI:** 10.1002/ccs3.70014

**Published:** 2025-04-14

**Authors:** Tayier Tuersong, Munire Shataer, Yan Chen, Gaosi Chen, Xiaoling Li, Linjie Lei, Ayiguli Younusi, Liangying Ma

**Affiliations:** ^1^ Department of Pharmacy Xinjiang Key Laboratory of Neurological Diseases Xinjiang Clinical Research Center for Nervous System Diseases Second Affiliated Hospital of Xinjiang Medical University Urumqi China; ^2^ Department of Histology and Embryology Basic Medical College of Xinjiang Medical University Urumqi China; ^3^ Department of Nephrology Wuhan Children's Hospital Wuhan Maternal and Child Healthcare Center Tongji Medical College Huazhong University of Science & Technology Wuhan China

**Keywords:** CDH2, chemoresistance, esophageal cancer, extracellular vesicles, immune evasion, LncRNA HOTAIR, miR‐375

## Abstract

Chemoresistance and immune evasion remain significant barriers to effective esophageal cancer (EC) treatment. This study explores the mechanistic role of extracellular vesicles (EVs) delivering LncRNA HOTAIR in modulating these processes. Using transcriptomic profiling, LncRNA HOTAIR was identified as a critical factor in EC progression. Its interaction with miR‐375 was examined via luciferase reporter assays and RNA immunoprecipitation. Paclitaxel‐resistant EC cells were treated with EVs containing HOTAIR, and the functional impact on proliferation, migration, invasion, and immune response was assessed through in vitro and in vivo models. LncRNA HOTAIR in EVs enhanced paclitaxel resistance by suppressing miR‐375 and increasing CDH2 expression. Furthermore, HOTAIR promoted immune escape by upregulating PD‐L1, impairing T‐cell‐mediated cytotoxicity. These changes were validated in patient‐derived EC models. This study demonstrates that EV‐LncRNA HOTAIR mediates chemoresistance and immune evasion in EC by targeting the miR‐375/CDH2 axis. These findings provide a foundation for novel therapeutic interventions targeting EV‐HOTAIR.

## Graphical abstract

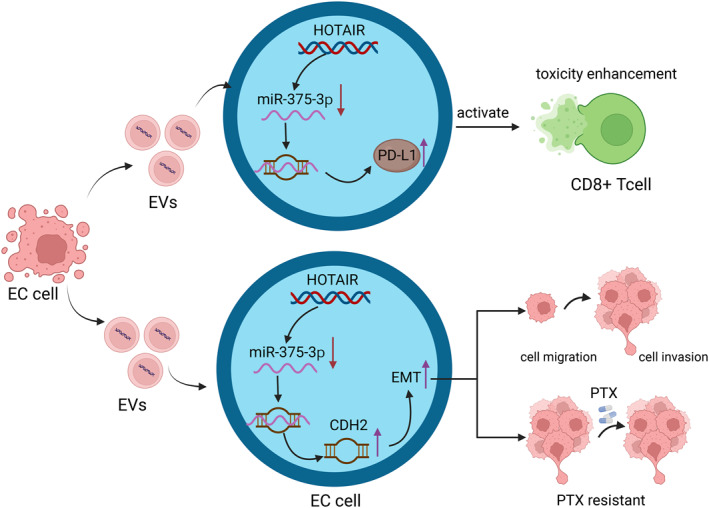

## 1 INTRODUCTION

Esophageal cancer (EC) is a common malignant tumor of the digestive tract with high incidence and mortality rates worldwide. EC is known for its high invasiveness and metastatic potential,^1^ making treatment difficult and prognosis poor.^2^, ^3^ Clinically, EC patients are typically treated with traditional methods such as surgery, chemotherapy, and radiotherapy but the effectiveness of these treatments is often compromised by factors such as chemotherapy resistance and immune evasion,^4^, ^5^ especially in advanced and recurrent patients paclitaxel (PTX) is a widely used chemotherapy drug in EC treatment but long‐term use leads to resistance in EC cells,^4^ decreasing its efficacy and increasing recurrence and mortality rates. Meanwhile, EC cells evade immune surveillance and clearance through various mechanisms^1^ allowing continuous growth and spread in the host. This resistance and immune evasion severely limit EC treatment effectiveness, highlighting the urgent need to explore its molecular mechanisms and identify new molecular targets to provide a theoretical basis for developing novel treatments.

Recent studies have shown that the tumor microenvironment plays a crucial role in EC's resistance and immune evasion processes.^6^ The intercellular communication mechanisms in the tumor microenvironment involve direct cell contact and molecular signaling through secreted substances such as cytokines and exosomes.^7^ Extracellular vesicles (EVs) are nanoscale vesicles secreted by cells, capable of transporting proteins, mRNA, microRNA (miRNA), long noncoding RNA (LncRNA), and other biomolecules ^8^ for long‐distance intercellular communication. Studies have shown that EVs play a significant role in cancer progression by delivering specific molecules to target cells, regulating gene expression in these cells, and affecting tumor growth, metastasis, drug resistance, and immune evasion.^9^ However, the mechanisms of action of specific molecules delivered by EVs in EC resistance and immune evasion were not fully studied, leaving significant gaps in our understanding of the molecular pathogenesis of EC.

In various cancers, LncRNAs have been found to be closely related to tumor progression and therapeutic resistance. Long noncoding RNA HOX transcript antisense intergenic RNA (LncRNA HOTAIR), that is highly expressed in several cancers,^10^ has gained increasing attention for its role in EC.^11^ HOX transcript antisense intergenic RNA (HOTAIR) regulates target gene expression by competitively binding to miRNAs,^12^ thereby playing a significant role in tumor cell proliferation, metastasis, and drug resistance. Previous studies have shown that high expression of HOTAIR in cancers, such as breast cancer and lung cancer, is closely associated with tumor invasiveness, drug resistance, and poor prognosis.^13^, ^14^ Additionally, HOTAIR has been found to upregulate the expression of key oncogenes by inhibiting the functional binding of miRNAs, providing a survival advantage to tumor cells. However, in EC, the specific mechanisms of HOTAIR's interaction with miRNAs and its delivery through EVs to target cells in drug resistance and immune evasion still need further verification and exploration. Therefore, in‐depth research on the mechanism of HOTAIR in EC, especially its delivery via EVs and its impact on miRNA regulation, is of significant importance for understanding the progression of EC.

This study aims to investigate the potential mechanism by which tumor cell‐derived EVs deliver LncRNA HOTAIR, contributing to PTX resistance and immune evasion in EC. By screening differentially expressed lncRNAs and constructing an lncRNA‐miRNA interaction network, this study focuses on the interaction between LncRNA HOTAIR and miR‐375. Through a combination of in vitro functional assays and EV isolation and characterization experiments, we will evaluate the role and regulatory pathways of HOTAIR in EC resistance and immune evasion. The findings of this study are expected to enhance the understanding of the molecular pathological mechanisms underlying EC, facilitate the identification of new molecular targets, and provide insights for future targeted therapeutic strategies. This is particularly relevant for EC patients exhibiting PTX resistance and immune evasion, potentially laying the foundation for novel intervention approaches.

## 2 MATERIALS AND METHODS

### 2.1 Data acquisition and processing

The dataset GSE111011 (Normal: *n* = 7, EC: *n* = 7) was downloaded from the GEO database (https://www.ncbi.nlm.nih.gov/geo/). Differential expression analysis of gene expression between EC and normal tissues was performed using the “limma” package, and significantly upregulated or downregulated genes were selected with the criteria of |log_2_ fold change| > 1 and *p*‐value <0.05. Differentially expressed lncRNAs were extracted for further analysis.

RNA count expression matrices corresponding to EC (TCGA‐ESCA) were downloaded from The Cancer Genome Atlas Program (TCGA) database (https://www.cancer.gov/ccg/research/genome‐sequencing/tcga). One metastatic sample was removed, resulting in 184 cancer samples and 13 normal samples in TCGA.

### 2.2 Survival analysis

Based on the HOTAIR expression levels of TCGA‐ESCA patients (*n* = 184), the cohort was divided into high and low‐expression groups. In survival analysis, stratification variables included different time points (1 year, 3 years, and 5 years) and pathological stages (Stage I, II, III, and IV and primary tumor stages T1, T2, T3, and T4). Kaplan–Meier survival analysis was performed to assess the survival differences between the groups, and the log‐rank test was used to determine the statistical significance of survival differences. Data analysis was conducted using the “survival” and “survminer” packages in R for survival curve plotting and statistical testing, comparing survival differences at various stages and time points.

### 2.3 Correlation analysis between miR‐375 and CDH2 expression

Based on TCGA‐ESCA data (*n* = 162), the correlation between miR‐375 and CDH2 expression was explored. First, the expression data of miR‐375 and CDH2 were log_2_ (TPM+1) normalized. Spearman correlation analysis was then used to calculate the correlation coefficient (R‐value) between the two. Correlation analysis and visualization were performed using R's “ggplot2” and “ggpubr” packages to create scatter plots.

### 2.4 ROC curve analysis of CDH2 for prognostic prediction

To evaluate the predictive prediction ability of CDH2 expression for patients (*n* = 163) at different time points (1 year, 3 years, and 5 years), we performed ROC (receiver operating characteristic) curve analysis to assess CDH2's predictive effectiveness at each time point. First, survival data and CDH2 expression levels from the TCGA‐ESCA database were collected and organized. Then, using the “survivalROC” package in R, ROC curves for 1‐year, 3‐year, and 5‐year survival were calculated, and the corresponding area under the curve (AUC) values were obtained as a measure of prediction accuracy.

### 2.5 ROC curve analysis of HOTAIR and TUSC7 for prognostic prediction

Based on TCGA‐ESCA data, the potential role of HOTAIR and TUSC7 in prognostic prediction for patients (*n* = 174) was evaluated. First, the expression data of HOTAIR and TUSC7 from the TCGA‐ESCA dataset were extracted, along with the corresponding survival information of the patients. Then, the “pROC” package in R was used to perform ROC curve analysis for the expression levels of HOTAIR and TUSC7, in order to calculate the AUC value as a measure of their predictive accuracy.

### 2.6 SVM and LASSO analysis

Biomarkers were selected using a combination of LASSO logistic regression and support vector machine recursive feature elimination (SVM‐RFE) algorithms. As a dimensionality reduction method, LASSO is used for variable selection and complexity adjustment when fitting generalized linear models. LASSO analysis was performed using the “glmnet” package (v4.1–8) with 10‐fold cross‐validation and penalty parameters. For the SVM‐RFE analysis, the “e1071” package (v1.7–14) was used with half above set to 50 and the k‐fold cross‐validation value set to 10.

### 2.7 Prediction of lncRNA‐miRNA and miRNA‐mRNA interactions

The RNADisease database (http://www.rnadisease.org/) was used to screen for differentially expressed miRNAs related to EC. The miRWalk database (http://mirwalk.umm.uni‐heidelberg.de/) was used to predict the target genes of the feature miRNAs.

### 2.8 Binding site prediction

The RNAhybrid tool predicted the binding sites between miRNAs and lncRNAs (parameters set to hits per target: 5, energy threshold: −20). The TargetScanHuman tool predicted the binding sites between miRNAs and mRNAs.

### 2.9 Cell culture

The EC cell line KYSE30 was purchased from Procell (CL‐0577), KYSE150 from Procell (CL‐0638), and CD8 T cells from Lonza (2W‐300). Cells were cultured in RPMI‐1640 medium (Gibco, USA) containing 10% fetal bovine serum (FBS) (Gibco, USA), 100 U/mL penicillin, and 100 μg/mL streptomycin (Thermo Fisher Scientific, USA), and incubated in a 37°C, 5% CO_2_ incubator. When the cells reached 70%–80% confluence, they were cultured for an additional 48 h in a serum‐free medium, and the supernatant was collected for EV isolation. CD8^+^ T cells were cultured in RPMI‐1640 medium containing 10% FBS, 100 U/mL penicillin, and 100 μg/mL streptomycin, and activated by KYSE30 or KYSE150 cell lysate, anti‐CD3e (10 μg/mL), and anti‐CD28 (2 μg/mL) for 48 h.

Co‐culture experiment: The treated KYSE30 or KYSE150 cells were co‐cultured with activated T cells at a 1:10 ratio for 48 h. The cell viability of KYSE30 or KYSE150 cells after co‐culture was measured using the CCK‐8 assay. ELISA was used to measure the levels of TNF‐α (550610, BD Biosciences, USA), IFN‐γ (88–7316‐88, Thermo Fisher Scientific, USA), IL‐2 (88–7025‐88, Thermo Fisher Scientific, USA), IL‐10 (BMS215/2TEN, Thermo Fisher Scientific, USA), IL‐1β (BMS224‐2, Thermo Fisher Scientific, USA), and TGF‐β (DY240, R&D Systems, USA) in the co‐culture medium.

### 2.10 Establishment and validation of PTX‐Resistant cell line

A 5 mM PTX (580555, Sigma‐Aldrich, USA) stock solution was prepared, dissolved in DMSO and further diluted in a culture medium for PTX treatment. The PTX‐resistant cell line was established in vitro by intermittently exposing KYSE30 or KYSE150 cells to high concentrations of PTX and gradually increasing the exposure time. When the cells reached 70%–80% confluence, 0.625 μg/mL of PTX was added to the culture medium for 2 h, followed by three washes with PBS. The cells were cultured in a fresh medium without PTX for 24 h. This process was repeated for 4–5 weeks, allowing surviving cells to proliferate and recover to a normal state. Subsequently, 0.625 μg/mL of PTX was added for 4 h, and the cycle was repeated thrice. Finally, the resistant cells were cultured in a medium without PTX for three generations and cryopreserved in liquid nitrogen to further validate PTX resistance by CCK‐8 assay.

### 2.11 Cell transfection and grouping

According to the manufacturer's instructions, gene transfection was performed using Lipofectamine 3000 transfection reagent (L3000001, Thermo Fisher Scientific (Invitrogen)). For transient transfection, approximately 2 × 10^4^ KYSE30 cells were seeded into a 96‐well plate and cultured until they reached 70%–80% confluence. Based on the cell density per well, an appropriate amount of plasmid (2–3 μg) was mixed with 2.5 μL of P3000 reagent in 125 μL of Opti‐MEM and gently pipetted to mix. Another 5 μL of Lipofectamine 3000 reagent was added to 125 μL of Opti‐MEM, mixed, and left to stand for 10 min. The two mixtures were combined to form a DNA‐Lipofectamine complex, incubated for 10–15 min, and then added to the cell culture wells, gently shaking the plate to ensure uniform distribution. The cells were incubated for 48 h at 37°C in a 5% CO_2_ incubator. All plasmids were purchased from Genepharma.

After 48 h of transfection, the fresh medium was replaced, and cells in each group were treated with 100 nM PTX for 48 h. The cells were divided into the following groups: (1) oe‐NC group, transfected with overexpression negative control plasmid; (2) oe‐HOTAIR group, transfected with overexpression HOTAIR plasmid (GenBank ID: NR_003716, Addgene, 26110); (3) miR‐375 miR‐NC group, transfected with miR‐NC plasmid; (4) miR‐375 mimic group, transfected with miR‐375 mimic plasmid (Dharmacon, mi0000783); (5) oe‐HOTAIR + miR‐375 mimic group, co‐transfected with HOTAIR and miR‐375 mimic plasmids; (6) PTX + si‐NC group, PTX treatment + transfected with silencing negative control plasmid; (7) PTX + EVs + si‐NC group, PTX treatment + EV treatment + transfected with silencing negative control plasmid; (8) PTX + EVs + si‐CDH2 group, PTX treatment + EV treatment + transfected with specific siRNA to silence CDH2 gene; (9) PTX + EVs‐si‐NC group, PTX treatment + EVs derived from the EC cell line in the si‐NC group; (10) PTX + EVs‐si‐HOTAIR + oe‐NC group, PTX treatment + EVs derived from si‐HOTAIR KYSE30 cells + transfected with overexpression negative control plasmid; and (11) PTX + EVs‐si‐HOTAIR + oe‐CDH2 group, PTX treatment + EVs derived from the EC cell line in the si‐HOTAIR group + transfected with overexpression CDH2 plasmid. After 48 h of transfection, the expression levels of HOTAIR and miR‐375 in each group were detected by RT‐qPCR to verify transfection efficiency. The silencing sequences are listed in Table S1.

### 2.12 EVs isolation

EVs were isolated and purified from the EC cell line supernatant. Cells were seeded into 10 cm culture dishes at a density of 1 × 10^6^ cells per dish and cultured in DMEM medium containing 10% EVs‐depleted FBS (System Biosciences). The cells were cultured at 37°C with 5% CO_2_ for 48 h, and the culture supernatant was collected for EV extraction.

The collected supernatant was centrifuged at 2000 g for 30 min at 4°C to remove cells and debris. The supernatant was then filtered through a 0.22 μm filter (Sigma Aldrich), and the filtrate was ultracentrifuged at 100,000 g at 4°C for 90 min using an Optima TLX ultracentrifuge (Beckman Coulter) to collect the EVs. The pellet (at the bottom of the centrifuge tube) was resuspended in phosphate‐buffered saline (PBS) and subjected to ultracentrifugation again under the same conditions. Finally, the EVs were washed with PBS and collected by ultracentrifugation at 100,000 g at 4°C for 90 min. The protein concentration of the purified EVs was determined using a NanoDrop spectrophotometer (Thermo Fisher Scientific), and the isolated EVs were used for further analysis.

### 2.13 NTA

NTA (Nanosight NS300, Malvern, UK) was used to determine the particle size distribution of EVs. The EV suspension was diluted to an appropriate concentration (1:1000) and analyzed using the NTA system. The particle size distribution and concentration of the EVs were recorded, with three measurements per sample to ensure the reliability of the data.

### 2.14 Dil dye labeling and uptake observation of EVs

Dil dye (D3911, Thermo Fisher Scientific, USA) was used to label the isolated EVs. The EV suspension was mixed with Dil dye at a 1:200 ratio and incubated at 37°C for 30 min. After incubation, the unbound dye was removed by ultracentrifugation, and the labeled EVs were resuspended in PBS. The labeled EVs were then added to the PTX‐resistant EC cell line, incubated for 4 h, and washed three times with PBS to remove any uninternalized EVs. Fluorescence microscopy (Leica Microsystems, Germany) was used to observe and capture images of the EV uptake.

### 2.15 TEM

TEM (Hitachi H‐7650, Japan) was employed to observe the morphology of isolated EVs. A drop of the isolated EV suspension was placed onto a carbon‐coated copper grid, followed by negative staining with a 2% phosphotungstic acid solution (Sigma‐Aldrich, USA). After staining, TEM was used to observe the morphology and size of the EVs, and images were captured for further analysis.

### 2.16 Scratch assay

The scratch assay was used to assess the migration ability of cells in each group. Treated cells were seeded at an appropriate density in a 6‐well plate, ensuring that the cells would reach over 90% confluence post‐seeding. Once confluence exceeded 90%, a straight line was scratched across the cell monolayer using a sterile pipette tip. Detached cells were gently washed away with PBS, and a serum‐free medium was added to prevent excessive cell proliferation that could interfere with migration analysis. After 24 h, images of the scratch area were captured using an inverted microscope (Olympus, Japan), and the scratch closure rate was calculated using ImageJ software (NIH, USA) to evaluate cell migration capacity.

### 2.17 Transwell invasion assay

A transwell chamber (8 μm pore size, Corning, USA) coated with Matrigel (BD Biosciences, USA) was used for the invasion assay. 5 × 10^4^ treated KYSE30 cells were added to the serum‐free upper chamber, whereas the lower chamber contained medium with 10% FBS as a chemoattractant. After 24 h of incubation, cells that had not invaded through the membrane were removed, and cells that had passed through the membrane were fixed with 4% formaldehyde and stained with 0.1% crystal violet. The stained cells on the membrane were then imaged using an inverted microscope (Olympus, Japan), and cell counts were determined with ImageJ software to assess invasion ability.

### 2.18 CCK‐8 assay

A CCK‐8 assay (C0037, Beyotime, China) was performed to evaluate the proliferation and viability of cells in each group. Treated KYSE30 cells (including transfected cells, PTX‐resistant cells treated with EVs, and cells co‐cultured with T cells) were seeded at a density of 5000 cells/well in a 96‐well plate, with five replicates per group. Cells were incubated for 24, 48, 72, and 96 h. Cells were incubated immediately for 2 h post‐seeding for the T‐cell co‐culture assay. At each time, 10 μL of CCK‐8 reagent was added to each well, followed by a 2‐h incubation. After incubation, absorbance was measured at 450 nm using a microplate reader (BioTek, USA), and cell proliferation or viability was calculated based on the absorbance values.

### 2.19 TUNEL staining assay

To assess apoptosis in each group, treated KYSE30 cells were seeded in a 24‐well plate. After 48 h of culture, cells were stained using a TUNEL apoptosis detection kit (C1086, Beyotime, China) following the manufacturer's instructions. After cell fixation, labeled dUTP was added and incubated for 1 h. Cells were then stained with DAPI for nuclear visualization. Images were acquired using a fluorescence microscope (Leica Microsystems, Germany), and the proportion of TUNEL‐positive cells was calculated using ImageJ software.

### 2.20 RT‐qPCR

According to the experimental protocol, total RNA was extracted from tissues and cells using TRIzol reagent (15596026, Invitrogen, USA) following the manufacturer's instructions. For miRNA expression analysis, a polyadenylation method was used. The All‐in‐One™ miRNA qRT‐PCR Detection Kit (AOMD‐Q100, GeneCopoeia) was employed, wherein miRNA was polyadenylated at the 3′ end, followed by reverse transcription using specific primers. Quantitative PCR was performed using SYBR Green dye to detect specific miRNA levels with U6 as an internal control. For lncRNA and mRNA detection, the PrimeScript RT reagent kit (Takara) was used to reverse transcribe RNA to cDNA, followed by RT‐qPCR using SYBR Premix Ex Taq II (Takara), with GAPDH as an internal control. Results were calculated using the 2^−ΔΔCT^ method. Primer sequences are detailed in Table S2.

### 2.21 Western blot (WB)

WB was performed to detect and validate the expression levels of various proteins in the experiment. Total protein from cells or EVs was extracted using RIPA lysis buffer (89901, Thermo Fisher Scientific, USA), and protein concentration was measured using a BCA Protein Assay Kit (P0011, Beyotime, China). For electrophoresis, 20–30 μg of protein was loaded onto a 10% or 12% SDS‐PAGE gel. After electrophoresis, proteins were transferred to a PVDF membrane (FFP19, Beyotime, China) using the wet transfer method. The membrane was blocked with 5% skim milk (P0216, Beyotime, China) at room temperature for 1 h to reduce nonspecific binding. After blocking, the membrane was incubated overnight at 4°C with specific primary antibodies (antibody concentrations are listed in Table S3). After washing with PBS, the membrane was incubated with an HRP‐conjugated secondary antibody at room temperature for 1 h. Protein signals were developed using ECL substrate (Thermo Fisher Scientific, USA) and detected with the ChemiDoc MP imaging system (Bio‐Rad, USA) with β‐actin as the loading control.

### 2.22 Dual‐luciferase reporter assay

A dual‐luciferase reporter assay was conducted to verify the interaction between LncRNA HOTAIR and miR‐375 and between miR‐375 and CDH2. The 3′UTR fragments of HOTAIR and CDH2 were cloned into the pGL3 vector (Bjlalb, China) to construct wild‐type (WT) and mutant (MUT) reporter plasmids. These plasmids were then co‐transfected with miR‐375 mimics or miR NC molecules and the pRL‐TK vector (Promega, USA) containing the Renilla luciferase gene for normalization, into KYSE30 cells. After 48 h of transfection, the luciferase activities of the firefly and Renilla were measured using the Dual‐Luciferase Reporter Assay System (E1910, Promega, USA). The ratio of firefly to Renilla luminescence represented the relative luciferase activity.

### 2.23 RNA pull‐down assay

To investigate the interaction between LncRNA HOTAIR and miR‐375, an RNA pull‐down assay was performed. Biotin‐labeled miR‐375 probes (339351, Qiagen, Germany) were transfected into KYSE30 cells. After 24 h, cells were lysed using RIPA lysis buffer, and the lysates were subjected to pull‐down using streptavidin magnetic beads (Thermo Fisher Scientific, USA). The bound RNA was then extracted using TRIzol (Invitrogen, USA), and the enrichment of HOTAIR was analyzed by RT‐qPCR.

### 2.24 Statistical analysis

All experimental data were processed using GraphPad Prism 8.0. An unpaired *t*‐test was used to compare data between two groups, whereas one‐way ANOVA was used for multiple group comparisons. Levene's test was used to assess the homogeneity of variance. If variances were equal, Dunnett's *t*‐test and LSD‐t test were employed for pairwise comparisons; if variances were unequal, Dunnett's T3 test was used. A *p*‐value <0.05 was considered statistically significant.

## 3 RESULTS

### 3.1 Potential mechanism by which LncRNA HOTAIR regulates CDH2 expression via miR‐375 to promote EC progression

**FIGURE 1:**
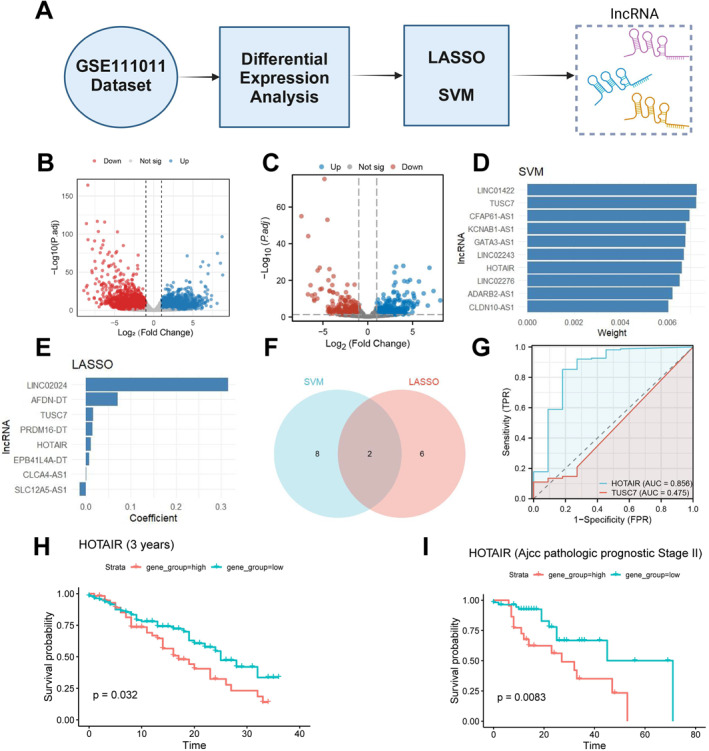
Screening of Key LncRNAs. (A) Flowchart for screening key LncRNAs; (B) volcano plot of differential expression in the GSE111011 dataset, showing significantly upregulated and downregulated genes (Normal: *n* = 7, EC: *n* = 7); (C) volcano plot of lncRNA expression in EC and normal tissues with blue and red indicating significantly downregulated and upregulated lncRNAs, respectively (Normal: *n* = 7, EC: *n* = 7); (D) SVM analysis showing the top 10 characteristic LncRNAs ranked by weight; (E) LASSO regression analysis showing selected LncRNAs and their corresponding regression coefficients; (F) Venn diagram of intersecting results from SVM and LASSO analyses; (G) ROC curves from TCGA database samples demonstrating the classification ability of intersecting LncRNAs (HOTAIR and TUSC7), with AUC values assessing model performance; (H) comparison of 3‐year truncated survival between high and low HOTAIR expression (high vs. low) in TCGA database samples; and (I) overall survival comparison between high and low HOTAIR expression (high vs. low) in Stage II samples from the TCGA database.

To explore the potential role of lncRNAs in EC, differential expression analysis was performed on dataset GSE111011, followed by feature lncRNA selection using LASSO regression and an SVM model (Figure 1A). The differential expression analysis revealed a total of 2677 significantly upregulated genes (red) and 3341 downregulated genes (blue) in EC and normal tissues (Figure 1B), laying a foundation for further lncRNA screening. Upon isolating the lncRNAs from GSE111011, the analysis showed expression differences of lncRNAs between EC and normal groups, with 283 significantly upregulated lncRNAs and 304 downregulated (Figure 1C), indicating that some lncRNAs might play crucial regulatory roles in EC.

To identify the most promising feature of lncRNAs, SVM and LASSO regression analyses were applied to 587 differentially expressed lncRNAs. SVM analysis identified the top 10 feature lncRNAs as LINC01422, TUSC7, CFAP61‐AS1, KCNAB1‐AS1, GATA3‐AS1, LINC02243, HOTAIR, LINC02276, ADARB2‐AS1, and CLDN10‐AS1, suggesting their potential significance in EC (Figure 1D). Concurrently, LASSO regression further refined the selection of feature lncRNAs by compressing and selecting significant coefficients, highlighting LINC02024, AFDN‐DT, TUSC7, PRDM16‐DT, HOTAIR, EPB41L4A‐DT, and CLCA4‐AS1 as positive contributors, implying their promotive role in EC (Figure 1E). Integrating results from both methods, the intersection of selected lncRNAs was identified with HOTAIR and TUSC7 emerging as shared feature lncRNAs in both analyses (Figure 1F). ROC curve analysis of these intersecting lncRNAs was conducted to evaluate their classification ability; results indicated that HOTAIR had an AUC value of 0.856, whereas TUSC7's AUC was 0.475, suggesting that HOTAIR has stronger classification potential, hence selected for further research (Figure 1G).

To investigate the association of HOTAIR expression with TNM staging and other clinical characteristics in EC patients, survival analysis was conducted across different time points (1‐year, 3‐year, and 5‐year survival) and various pathological stages (Overall Stages I, II, III, and IV, and T stages: T1, T2, T3, and T4 indicating tumor size and extent). Results showed that patients with high HOTAIR expression had significantly reduced 3‐year survival (Figure 1H). Within Stage II cohorts, high HOTAIR expression was associated with a marked reduction in overall survival (Figure 1I).

**FIGURE 2:**
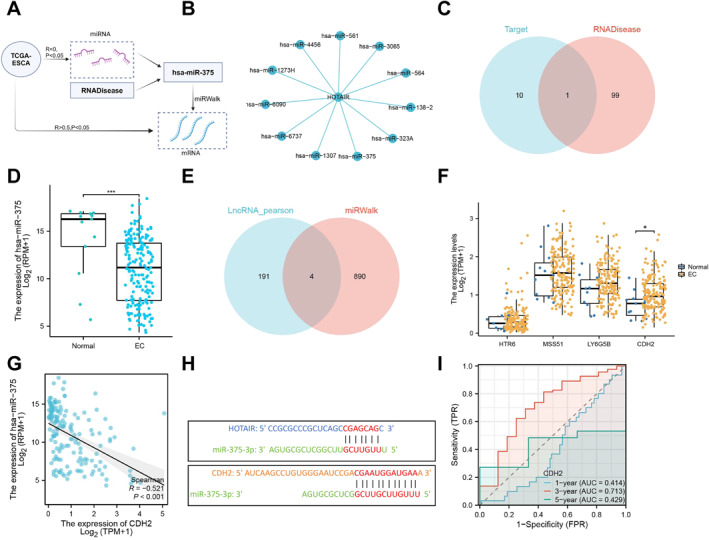
Regulatory Network of HOTAIR in EC and Expression Analysis of Its Target miRNA and mRNA. (A) Flowchart for screening key miRNAs and mRNAs; (B) miRNA‐target network of HOTAIR and its related miRNAs; (C) Venn diagram analysis of miRNAs identified from RNADisease database and selected miRNAs; (D) expression levels of hsa‐miR‐375 in EC and normal tissues from TCGA database (Normal: *n* = 13, EC: *n* = 184); (E) Venn diagram analysis of HOTAIR‐related mRNAs and hsa‐miR‐375 target mRNAs; (F) expression comparison of intersecting mRNAs between EC and normal tissues in TCGA database; (G) correlation analysis of hsa‐miR‐375 and CDH2 in TCGA database samples; (H) predicted binding sites of HOTAIR and CDH2 with miR‐375 using RNAhybrid tool; and (I) survival prediction capability of CDH2 expression for EC patients at different time points (1‐year, 3‐year, 5‐year) in TCGA database with blue, red, and green curves representing 1‐year, 3‐year, and 5‐year survival prediction curves, respectively.

To further explore the miRNA network associated with HOTAIR in EC, RNA‐seq data from the TCGA‐ESCA cohort was downloaded and analyzed. Based on these data, correlation analysis between HOTAIR and miRNAs identified 11 significantly negatively correlated with HOTAIR (*R* < 0, *p* < 0.05) (Figure 2A). The results presented the interaction network of HOTAIR with multiple miRNAs, constructing a miRNA‐target network (Figure 2B), indicating that these miRNAs may play key roles in regulating HOTAIR's functions in EC.

The top 100 differentially expressed miRNAs in EC were obtained from the RNADisease database and intersected with the previously identified miRNAs to further identify key miRNAs in EC. The analysis revealed that hsa‐miR‐375 is the only miRNA significantly associated with HOTAIR and differentially expressed in EC, making it the primary candidate for subsequent functional studies (Figure 2C).

Based on the TCGA‐ESCA dataset, we examined the expression level of hsa‐miR‐375 in EC, finding that hsa‐miR‐375 is significantly downregulated in EC tissues. This downregulation may be associated with EC progression as miR‐375 is thought to function as a tumor suppressor under normal conditions. Reducing its expression could lead to uncontrolled expression of certain oncogenes, thereby promoting cancer progression (Figure 2D). To explore the regulatory mechanism between HOTAIR and hsa‐miR‐375, we further analyzed the TCGA database and identified 195 mRNAs associated with HOTAIR. Additionally, miRWalk was used to predict 894 target mRNAs of hsa‐miR‐375. The intersection of these datasets identified four mRNAs (HTR6, MSS51, LYG6SB, CDH2) (Figure 2E). The expression levels of these four intersecting mRNAs in EC samples were evaluated using the TCGA‐ESCA dataset, revealing that CDH2 expression is significantly higher in EC tissues compared to normal tissues (Figure 2F). Furthermore, an analysis of the relationship between hsa‐miR‐375 and CDH2 expression showed a significant negative correlation (*R* = −0.521, *p* < 0.001), suggesting that decreased expression of hsa‐miR‐375 tends to coincide with increased CDH2 expression (Figure 2G). Finally, the RNAhybrid tool was used to predict binding sites between HOTAIR, CDH2, and hsa‐miR‐375‐5p/hsa‐miR‐375. The results indicate partial pairing between miR‐375‐3p and the sequences of both HOTAIR and CDH2, suggesting a competitive relationship between HOTAIR and CDH2 for binding to miR‐375 (Figure 2H). This implies that the abnormal expression of CDH2 in EC may be co‐regulated by HOTAIR and hsa‐miR‐375. Using the TCGA‐ESCA dataset, we assessed the performance of 1‐year, 3‐year, and 5‐year survival prediction models based on CDH2 expression. The results showed that the 3‐year survival prediction model had an AUC of 0.713, indicating moderate predictive value, while the 1‐year (AUC = 0.414) and 5‐year (AUC = 0.429) predictions were less effective, approaching random classification (Figure 2I).

This study identified HOTAIR as a key lncRNA in EC significantly associated with miR‐375. Further analysis suggests that HOTAIR and miR‐375 may jointly regulate the expression of the downstream target CDH2 which is significantly upregulated in EC tissues compared to normal tissues. Collectively, the regulatory axis formed by HOTAIR, miR‐375, and CDH2 may play a crucial role in the initiation and progression of EC.

### 3.2 LncRNA HOTAIR enhances EC cell proliferation, migration, and invasion via miR‐375/CDH2 axis regulation

**FIGURE 3:**
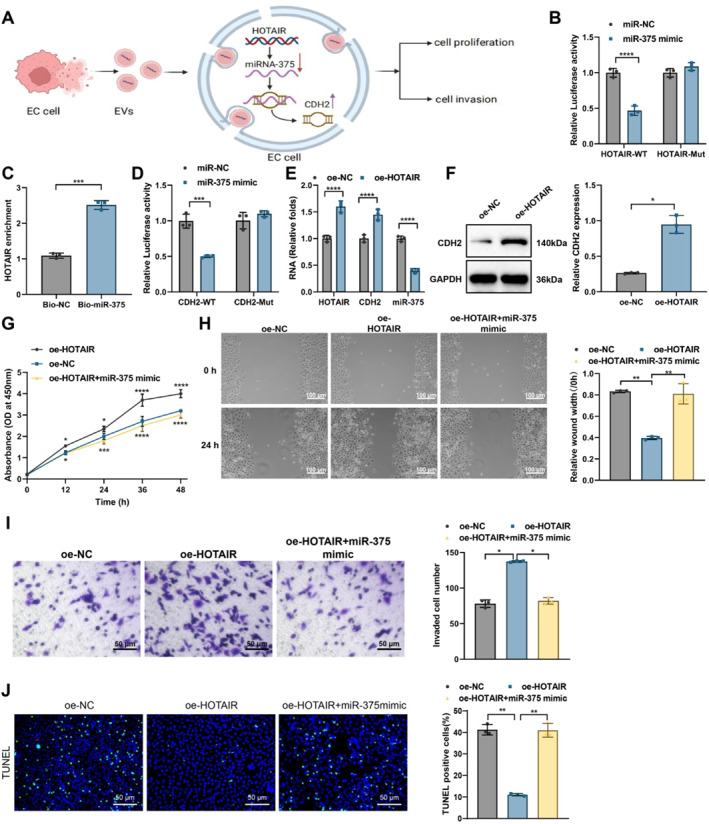
Validation and functional phenotyping of HOTAIR‐miR‐375/CDH2 axis interaction. (A) Schematic illustration of the effect of LncRNA HOTAIR on EC cell proliferation and invasion via miR‐375/CDH2 axis; (B) dual‐luciferase reporter assay showing the impact of HOTAIR overexpression on miR‐375 luciferase activity in KYSE30 cells; (C) RNA pull‐down assay evaluating HOTAIR‐mediated miR‐375 enrichment; (D) dual‐luciferase reporter assay detecting the effect of miR‐375 mimic transfection on CDH2 3′UTR luciferase activity; (E) RT‐qPCR detecting mRNA expression levels of HOTAIR, miR‐375, and CDH2; (F) WB detecting CDH2 protein expression; (G) CCK‐8 assay assessing cell proliferation; (H) wound healing assay evaluating cell migration (bar: 100 μm); (I) transwell invasion assay detecting cell invasion (bar: 50 μm); and (J) TUNEL staining detecting cell apoptosis rates (bar: 20 μm). Cell experiments were repeated three times. * indicates comparisons between groups, *p* < 0.05, **p < 0.01, ***p < 0.001, ****p < 0.001.

We conducted a series of experiments to investigate the effect of LncRNA HOTAIR on the proliferation and invasion capacity of EC cells through the miR‐375/CDH2 axis (Figure 3A). The interaction between LncRNA HOTAIR and miR‐375 was validated by a dual‐luciferase reporter and RNA pull‐down assays. Results showed that in the KYSE30 cell line, overexpression of miR‐375 led to a significant reduction in luciferase activity in the HOTAIR‐WT group (Figure 3B). The RNA pull‐down assay further confirmed this interaction, showing that LncRNA HOTAIR could effectively enrich miR‐375 with an enrichment efficiency 2.5 times higher than the control group (Figure 3C). After transfection with miR‐375 mimics, luciferase activity in the CDH2 3′UTR region was significantly reduced, indicating that miR‐375 can directly target and downregulate CDH2 expression (Figure 3D). Additionally, RT‐qPCR results showed that in the HOTAIR‐overexpressing group, mRNA levels of HOTAIR and CDH2 significantly increased, whereas miR‐375 expression notably decreased (Figure 3E). Correspondingly, WB analysis demonstrated that CDH2 protein levels were significantly higher in the oe‐HOTAIR group compared to the control (Figure 3F).

The CCK‐8 assay results indicated that cell proliferation was significantly enhanced in the HOTAIR‐overexpressing cells, whereas cotransfection with miR‐375 mimics decreased proliferation capacity (Figure 3G). Furthermore, the scratch assay and transwell invasion assay results showed that HOTAIR overexpression markedly enhanced cell migration and invasion abilities; however, this effect was partially inhibited upon cotransfection with miR‐375 mimics (Figure 3H–I). The TUNEL staining results revealed that the apoptosis rate was significantly reduced in the HOTAIR overexpression group, whereas cotransfection with miR‐375 mimics led to an increase in apoptosis rate, although it remained lower than the control group (Figure 3J).

### 3.3 Successful isolation and verification of LncRNA HOTAIR in EVs derived from EC cells

**FIGURE 4:**
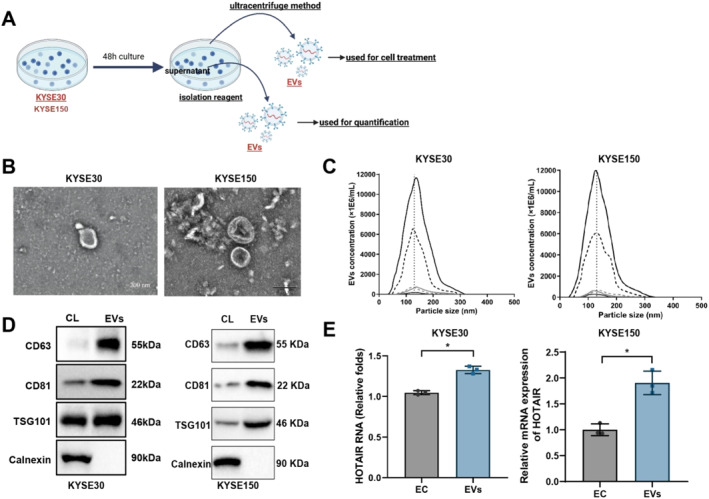
Characterization of EVs from KYSE30 and KYSE150 EC Cell lines and detection of HOTAIR. (A) Schematic diagram of EV isolation; (B) TEM observing the morphology of isolated EVs (bar: 200 nm); (C) NTA measuring the particle size distribution of isolated EVs; (D) WB detecting markers CD63, CD81, TSG101, and the negative marker Calnexin in isolated EVs; and (E) RT‐qPCR detecting HOTAIR expression levels in isolated EVs. Cell experiments were repeated three times. * indicates comparisons between groups, *p* < 0.05. CL: Cell lysate.

In this study, we successfully isolated EVs from the culture supernatant of the EC cell lines KYSE30 and KYSE150 (Figure 4A–B). TEM revealed that the EVs exhibited a typical spherical morphology with diameters ranging from approximately 100 to 150 nm (Figure 4B). NTA further indicated that the size distribution of EVs was primarily between 100 and 200 nm, with an average diameter of 130 nm, confirming a relatively uniform size distribution of the isolated EVs (Figure 4C). These results validate that the isolated EVs display typical morphological and size characteristics.

WB analysis showed that EV markers CD63, CD81, and TSG101 were prominently expressed in the isolated EVs, whereas the negative marker calnexin was undetectable (Figure 4D), indicating a high purity of the isolated EVs. Additionally, RT‐qPCR results demonstrated that the expression level of LncRNA HOTAIR was significantly higher in EVs compared to the cells, confirming that LncRNA HOTAIR is indeed enriched in EVs secreted by EC cells (Figure 4E). These findings establish a foundation for further investigation into the functional role of EV‐mediated LncRNA HOTAIR in EC.

### 3.4 EV‐mediated miR‐375/CDH2 axis promotes PTX resistance in EC cells, reversible by CDH2 inhibition

**FIGURE 5:**
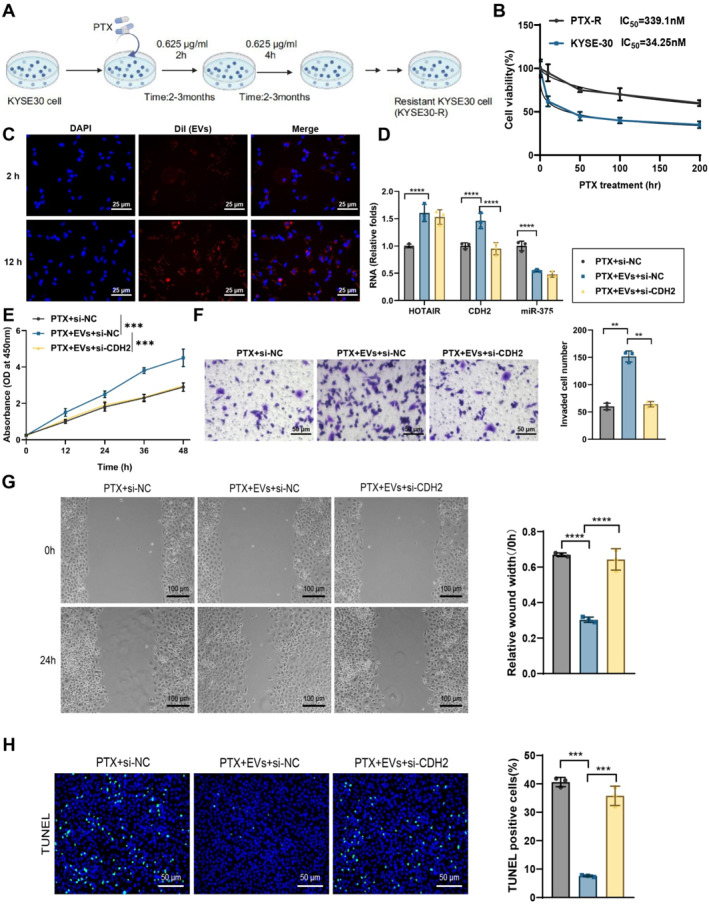
Effect of EVs‐mediated miR‐375/CDH2 axis on PTX resistance in KYSE30 cells. (A) Flowchart of constructing PTX‐resistant EC cell line KYSE30; (B) CCK‐8 assay evaluating the survival rate of resistant and sensitive cell lines after PTX treatment; (C) fluorescence microscopy showing the uptake efficiency of Dil‐labeled EVs by PTX‐resistant KYSE30 cells; (D) RT‐qPCR detecting mRNA expression levels of HOTAIR, miR‐375, and CDH2 in resistant cells; (E) CCK‐8 assay evaluating the survival rate of resistant cells in different treatment groups; (F) transwell migration assay assessing migration ability of resistant cells in different treatment groups (bar: 50 μm); (G) wound healing assay evaluating migration ability of resistant cells in different treatment groups (bar: 100 μm); and (H) TUNEL staining detecting apoptosis rates of resistant cells in different treatment groups. Cell experiments were repeated three times. * indicates comparisons between groups, *p* < 0.05, **p < 0.01, ***p < 0.001, ****p < 0.001.

In this study, we successfully established a PTX‐resistant EC cell line (Figures 5A, and S1A), and confirmed its resistance using the CCK‐8 assay. Results showed a significantly higher survival rate in the PTX‐resistant cell line than the PTX‐sensitive cells upon PTX treatment, validating the successful establishment of the PTX‐resistant model (Figures 5B and S1B). Next, isolated EVs were labeled with Dil dye, and fluorescence microscopy confirmed the efficient uptake of these EVs by EC cells with fluorescence intensity increasing over time (Figure 5C and S1C). The PTX‐resistant KYSE30 cells were divided into three groups: PTX + si‐NC, PTX + EVs + si‐NC, and PTX + EVs + si‐CDH2. The KYSE150 drug‐resistant cell line was grouped in the same manner as KYSE30. To determine the most effective siRNA for silencing CDH2, RT‐qPCR and WB analyses showed that both si‐CDH2‐1 and si‐CDH2‐2 significantly reduced CDH2 expression in KYSE30 and KYSE150 cells compared to si‐NC with si‐CDH2‐2 (referred to as si‐CDH2) showing a more pronounced effect; this sequence was used for subsequent experiments (Figure S2).

RT‐qPCR results indicated that, compared to the PTX + si‐NC group, the PTX + EVs + si‐NC group had significantly higher expression levels of HOTAIR and CDH2 in KYSE30 and KYSE150 cells, whereas miR‐375 expression was significantly decreased. In contrast, in the PTX + EVs + si‐CDH2 group, CDH2 expression was markedly reduced compared to the PTX + EVs + si‐NC group, whereas HOTAIR and miR‐375 levels showed no significant changes (Figures 5D and S1D). These results suggest that EVs‐HOTAIR upregulates CDH2 expression by sequestering miR‐375.

The CCK‐8 assay was used to evaluate cell proliferation across different treatment groups. Results showed a significant increase in cell survival in the PTX + EVs + si‐NC group compared to the PTX + si‐NC group, whereas cotransfection with si‐CDH2 resulted in a marked decrease in cell survival in the PTX + EVs + si‐CDH2 group compared to the PTX + EVs + si‐NC group (Figures 5E and S1E). Transwell invasion assays were performed to assess the invasive capacity of cells in each group, revealing that the PTX + EVs + si‐NC group exhibited significantly enhanced invasion compared to the PTX + si‐NC group in KYSE30 and KYSE150 cells. However, invasion capacity was notably reduced in the PTX + EVs + si‐CDH2 group compared to the PTX + EVs + si‐NC group (Figures 5F and S1F). This difference in migration ability was further validated by scratch assays where the PTX + EVs + si‐NC group showed a significant increase in migration compared to the PTX + si‐NC group in KYSE30 and KYSE150 cells, whereas cotransfection with si‐CDH2 resulted in a substantial reduction in migration in the PTX + EVs + si‐CDH2 group (Figure 5G and S1G). TUNEL staining assays were used to assess apoptosis. Results showed that apoptosis rates significantly decreased in the PTX + EVs + si‐NC group compared to the PTX + si‐NC group in KYSE30 and KYSE150 cells, whereas co‐transfection with si‐CDH2 led to a marked increase in apoptosis in the PTX + EVs + si‐CDH2 group compared to the PTX + EVs + si‐NC group (Figures 5H and S1H).

These findings suggest that EVs‐HOTAIR promotes PTX resistance by upregulating CDH2, and that inhibition of CDH2 attenuates this resistance effect.

### 3.5 EV‐LncRNA HOTAIR via miR‐375/CDH2 axis promotes PTX resistance in EC cells

**FIGURE 6:**
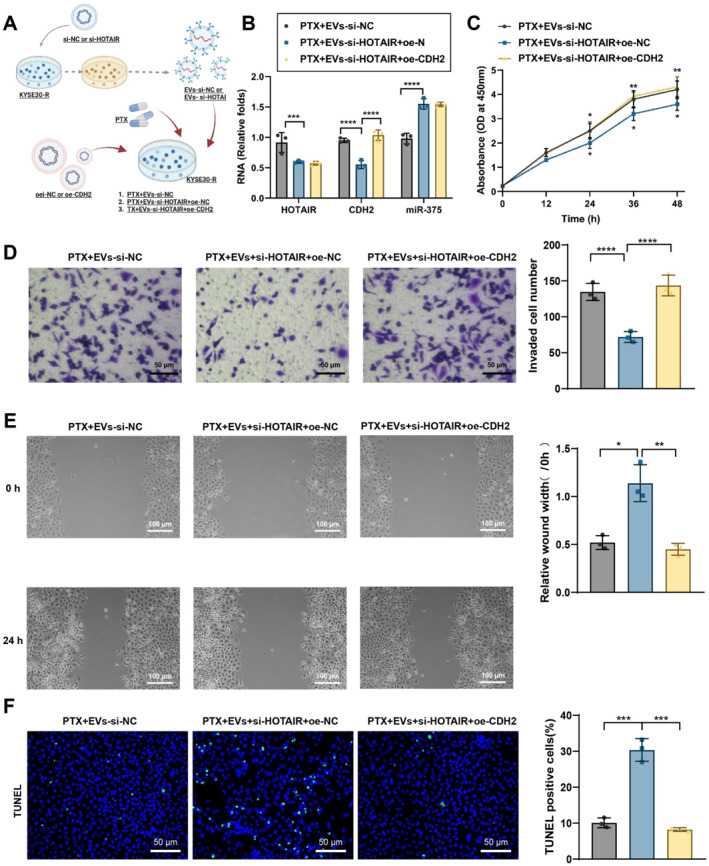
The effect of EV‐delivered LncRNA HOTAIR on PTX resistance in KYSE30 cells via the miR‐375/CDH2 axis. (A) Flowchart of EVs‐HOTAIR mediating the miR‐375/CDH2 axis and PTX resistance; (B) RT‐qPCR detecting expression levels of HOTAIR, CDH2, and miR‐375; (C) CCK‐8 assay evaluating the survival rate of KYSE30 cells in each group; (D) transwell migration assay assessing invasion ability of KYSE30 cells in each group; (E) wound healing assay evaluating migration ability of KYSE30 cells; and (F) TUNEL staining detecting apoptosis rates of KYSE30 cells in each group.

First, the efficacy of HOTAIR silencing and CDH2 overexpression was assessed. RT‐qPCR results showed that si‐HOTAIR‐1 significantly reduced HOTAIR expression compared to the si‐NC group in KYSE30 and KYSE150 cells (Figure S3A); thus, si‐HOTAIR‐1 (referred to as si‐HOTAIR) was used for subsequent experiments. Likewise, CDH2 expression was markedly increased in the oe‐CDH2 group compared to the oe‐NC group (Figure S3B). To confirm that EVs exert downstream effects by delivering LncRNA HOTAIR, EVs were isolated from EC cells transfected with either si‐NC or si‐HOTAIR (termed EVs‐si‐NC and EVs‐si‐HOTAIR, respectively). PTX‐resistant cancer cells were then divided into three groups: PTX + EVs‐si‐NC, PTX + EVs‐si‐HOTAIR + oe‐NC, and PTX + EVs‐si‐HOTAIR + oe‐CDH2 (Figures 6A and S4A).

RT‐qPCR results demonstrated that, compared to the PTX + EVs‐si‐NC + oe‐NC group in KYSE30 and KYSE150 cells, the PTX + EVs‐si‐HOTAIR + oe‐NC group exhibited significantly reduced HOTAIR and CDH2 expression with a significant increase in miR‐375 expression. In the PTX + EVs‐si‐HOTAIR + oe‐CDH2 group, CDH2 expression was significantly elevated compared to the PTX + EVs‐si‐HOTAIR + oe‐NC group, whereas HOTAIR and miR‐375 levels remained unchanged (Figures 6B and S4B). These findings suggest that EVs‐HOTAIR upregulates CDH2 expression by sequestering miR‐375.

The CCK‐8 assay indicated that cell viability was significantly reduced in the PTX + EVs‐si‐HOTAIR + oe‐NC group compared to the PTX + EVs‐si‐NC group in KYSE30 and KYSE150 cells. In contrast, cell viability was significantly increased in the PTX + EVs‐si‐HOTAIR + oe‐CDH2 group compared to the PTX + EVs‐si‐HOTAIR + oe‐NC group (Figures 6C and S4C). Transwell invasion assays showed that the invasive capacity of cells was significantly reduced in the PTX + EVs‐si‐HOTAIR + oe‐NC group compared to the PTX + EVs‐si‐NC group in KYSE30 and KYSE150 cells, whereas invasion was markedly enhanced in the PTX + EVs‐si‐HOTAIR + oe‐CDH2 group compared to the PTX + EVs‐si‐HOTAIR + oe‐NC group (Figures 6D and S4D). The scratch assay confirmed these migration trends, with the PTX + EVs‐si‐HOTAIR + oe‐NC group showing reduced migration compared to the PTX + EVs‐si‐NC group in KYSE30 and KYSE150 cells, and migration significantly increased in the PTX + EVs‐si‐HOTAIR + oe‐CDH2 group relative to the PTX + EVs‐si‐HOTAIR + oe‐NC group (Figures 6E and S4E). TUNEL staining assays evaluated apoptosis. Results showed that the apoptosis rate was significantly increased in the PTX + EVs‐si‐HOTAIR + oe‐NC group compared to the PTX + EVs‐si‐NC group in KYSE30 and KYSE150 cells, whereas apoptosis was markedly decreased in the PTX + EVs‐si‐HOTAIR + oe‐CDH2 group compared to the PTX + EVs‐si‐HOTAIR + oe‐NC group (Figures 6F and S4F).

These results indicate that EVs‐si‐HOTAIR inhibits PTX resistance by downregulating CDH2, whereas CDH2 overexpression significantly reverses this resistance effect.

### 3.6 EV‐mediated upregulation of programmed death‐ligand 1 (PD‐L1) promotes EC immune evasion

**FIGURE 7:**
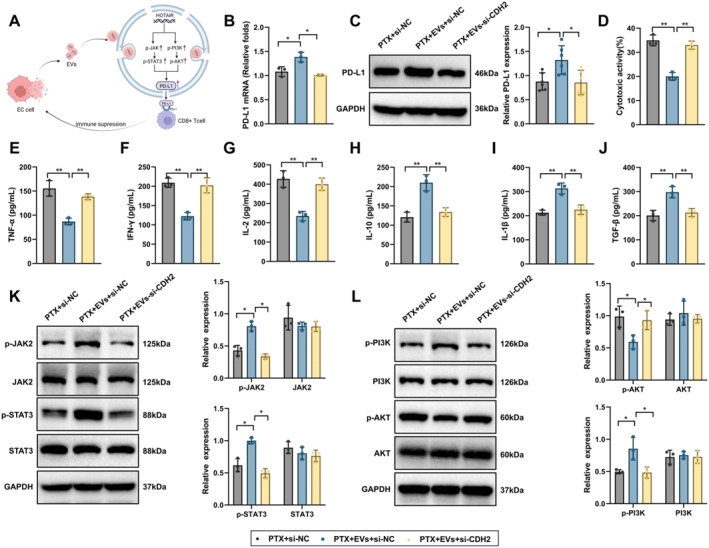
Study on the EVs‐mediated immune evasion mechanism. (A) Schematic diagram of the EVs‐mediated immune evasion mechanism in KYSE30; (B) RT‐qPCR detecting mRNA expression levels of PD‐L1 in different treatment groups of KYSE30 cells; (C) WB detecting protein expression levels of PD‐L1 in different treatment groups of KYSE30 cells; (D) CCK‐8 assay evaluating T cell‐mediated cytotoxicity against KYSE30 cells in different treatment groups; (E–J) ELISA detecting levels of TNF‐α, IFN‐γ, IL‐2, IL‐10, IL‐1β, and TGF‐β in co‐culture media of different treatment groups; and (K–L) WB analyzing activation levels of JAK/STAT and PI3K/AKT signaling pathways. Cell experiments were repeated three times. * indicates comparisons between groups, *p* < 0.05, **p < 0.01.

To investigate the mechanism by which LncRNA HOTAIR mediates immune evasion in EC through EVs (Figures 7A and S5A), we first analyzed PD‐L1 expression in EC cells across different treatment groups using RT‐qPCR and WB. Results showed that compared to the PTX + si‐NC group, PD‐L1 mRNA and protein expression levels significantly increased in the PTX + EVs + si‐NC group in KYSE30 and KYSE150 cells. In contrast, PD‐L1 expression significantly reduced in the PTX + EVs‐si‐HOTAIR + oe‐CDH2 group compared to the PTX + EVs + si‐NC group (Figures 7B–C and S5B–C).

Next, EC cells from the different treatment groups were co‐cultured with T cells, and the T cell cytotoxicity against cancer cells was evaluated. CCK‐8 assay results indicated that T cell killing capacity significantly decreased in the PTX + EVs + si‐NC group compared to the PTX + si‐NC group in KYSE30 and KYSE150 cells, whereas T cell killing capacity was notably restored in the PTX + EVs‐si‐HOTAIR + oe‐CDH2 group compared to the PTX + EVs + si‐NC group (Figures 7D and S5D). Subsequently, ELISA assays were conducted to measure cytokine levels. Compared to the PTX + si‐NC group, the PTX + EVs + si‐NC group exhibited significantly reduced secretion of TNF‐α, IFN‐γ, and IL‐2, along with significantly increased secretion of IL‐1β, IL‐10, and TGF‐β. In the PTX + EVs‐si‐HOTAIR + oe‐CDH2 group, secretion of TNF‐α, IFN‐γ, and IL‐2 significantly increased, whereas IL‐1β, IL‐10, and TGF‐β levels significantly decreased compared to the PTX + EVs + si‐NC group (Figures 7E–J and S5E–J).

Additionally, WB analysis revealed that activation of the JAK/STAT and PI3K/AKT signaling pathways was significantly enhanced in the PTX + EVs + si‐NC group in KYSE30 and KYSE150 cells, whereas these pathways showed markedly reduced activation in the PTX + EVs‐si‐HOTAIR + oe‐CDH2 group (Figures 7K–L and S5K‐L). These results suggest that CDH2 inhibition can partially reverse the immune evasion effect mediated by EVs and restore the killing ability of T cells against EC cells.

### 3.7 EV‐LncRNA HOTAIR via miR‐375/CDH2 axis upregulates PD‐L1 to promote EC immune evasion

**FIGURE 8:**
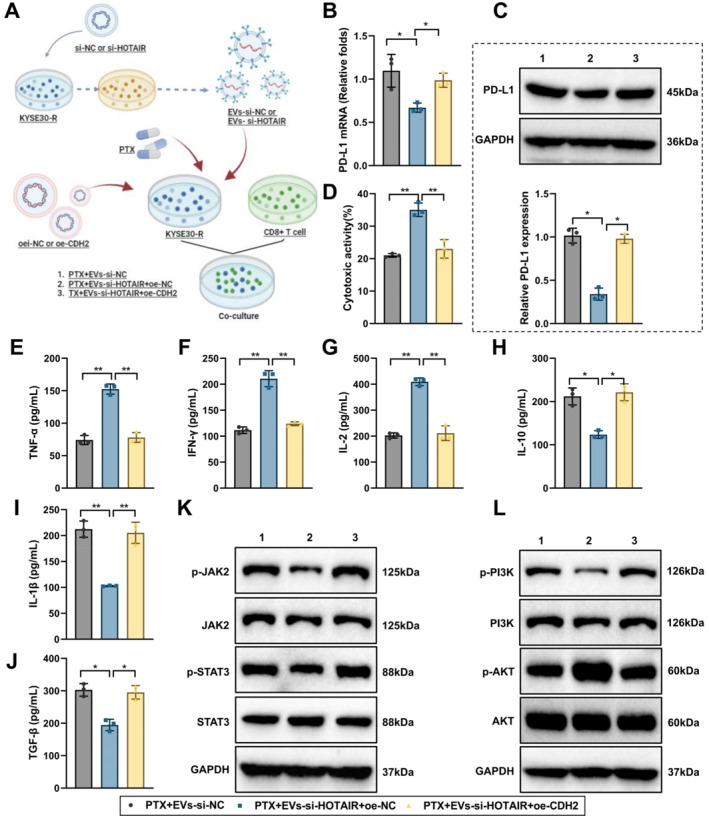
The effect of EV‐delivered LncRNA HOTAIR on immune evasion in KYSE30 cells via the miR‐375/CDH2 axis. (A) Experimental workflow of EVs‐LncRNA HOTAIR promoting immune evasion via the miR‐375/CDH2 axis; (B) RT‐qPCR detecting mRNA expression levels of PD‐L1 in different treatment groups of KYSE30 cells; (C) WB detecting protein expression levels of PD‐L1 in different treatment groups of KYSE30 cells; (D) CCK‐8 assay evaluating T cell‐mediated cytotoxicity against KYSE30 cells in different treatment groups; (E–J) ELISA detecting levels of TNF‐α, IFN‐γ, IL‐2, IL‐10, IL‐1β, and TGF‐β in co‐culture media of different treatment groups; and (K–L) WB analyzing activation levels of JAK/STAT and PI3K/AKT signaling pathways. Cell experiments were repeated three times. * indicates comparisons between groups, *p* < 0.05, **p < 0.01. Group 1 represents PTX + EVs‐si‐NC, Group 2 represents PTX + EVs‐si‐HOTAIR + oe‐NC, and Group 3 represents PTX + EVs‐si‐HOTAIR + oe‐CDH2.

To confirm that EVs exert downstream effects by delivering LncRNA HOTAIR, EVs were isolated from EC cells transfected with either si‐NC or si‐HOTAIR (termed EVs‐si‐NC and EVs‐si‐HOTAIR, respectively). PTX‐resistant cancer cells were divided into PTX + EVs‐si‐NC, PTX + EVs‐si‐HOTAIR + oe‐NC, and PTX + EVs‐si‐HOTAIR + oe‐CDH2. These groups were co‐cultured with activated CD8^+^ T cells (Figures 8A and S6A).

First, RT‐qPCR and WB assessed PD‐L1 expression levels in EC cells across treatment groups. Results showed that compared to the PTX + EVs‐si‐NC group in KYSE30 and KYSE150 cells, PD‐L1 mRNA and protein levels were significantly reduced in the PTX + EVs‐si‐HOTAIR + oe‐NC group in KYSE30 and KYSE150 cells. In contrast, PD‐L1 expression was significantly elevated in the PTX + EVs‐si‐HOTAIR + oe‐CDH2 group compared to the PTX + EVs‐si‐HOTAIR + oe‐NC group (Figures 8B–C and S6B–C).

Next, activated T cells were co‐cultured with EC cells from the different treatment groups to evaluate T cell cytotoxicity against cancer cells. The CCK‐8 assay results showed that T cell killing capacity significantly increased in the PTX + EVs‐si‐HOTAIR + oe‐NC group compared to the PTX + EVs‐si‐NC group in KYSE30 and KYSE150 cells, whereas T cell killing capacity notably reduced in the PTX + EVs‐si‐HOTAIR + oe‐CDH2 group compared to the PTX + EVs‐si‐HOTAIR + oe‐NC group (Figures 8D and S6D).

ELISA assays were then conducted to measure cytokine levels. Compared to the PTX + EVs‐si‐NC group in KYSE30 and KYSE150 cells, the PTX + EVs‐si‐HOTAIR + oe‐NC group exhibited significantly increased secretion of TNF‐α, IFN‐γ, and IL‐2, along with a marked decrease in IL‐1β, IL‐10, and TGF‐β secretion. In the PTX + EVs‐si‐HOTAIR + oe‐CDH2 group, the levels of TNF‐α, IFN‐γ, and IL‐2 significantly reduced, whereas IL‐1β, IL‐10, and TGF‐β significantly increased compared to the PTX + EVs‐si‐HOTAIR + oe‐NC group (Figures 8E–J and S6E–J).

Additionally, WB analysis indicated that the activation of JAK/STAT and PI3K/AKT signaling pathways significantly reduced in the PTX + EVs‐si‐HOTAIR + oe‐NC group in KYSE30 and KYSE150 cells, whereas these pathways were markedly activated in the PTX + EVs‐si‐HOTAIR + oe‐CDH2 group (Figures 8K–L and S6K–L). These findings suggest that overexpression of CDH2 can partially reverse the immune suppressive effects mediated by EVs‐si‐HOTAIR, restoring immune function against EC cells.

## 4 DISCUSSION

This study reveals the critical role of tumor cell‐derived EVs‐LncRNA HOTAIR in promoting immune evasion and PTX resistance in EC. We found that HOTAIR upregulates CDH2 expression by sequestering miR‐375, enhancing immune evasion and PTX resistance in EC cells. This discovery provides new insights into the role of EVs in the EC microenvironment and further confirms the regulatory potential of LncRNAs in cancer progression. Unlike previous research, this study is the first to propose a molecular mechanism where EVs‐LncRNA HOTAIR drives immune evasion and drug resistance in EC by regulating CDH2 expression via miR‐375. This finding enriches our understanding of the molecular pathology of EC and provides new potential targets for precise therapies, bearing significant scientific and clinical implications.

Previous studies have shown that high expression of LncRNA HOTAIR is closely related to tumor progression, drug resistance, and immune evasion across various cancers.^15^, ^16^, ^17^ For example, in breast cancer, HOTAIR has been shown to upregulate certain oncogenes by sequestering miRNAs,^18^ thus promoting tumor cell proliferation and metastasis.^19^ Building upon these insights, our study extends the mechanism of HOTAIR to EC, revealing that it regulates CDH2 expression in EVs through miR‐375. Previous research has focused on interactions between HOTAIR and other miRNAs such as miR‐141 and miR‐218.^20^ This study is the first to identify that HOTAIR acts through miR‐375, shedding light on HOTAIR's regulatory role in the specific EC microenvironment. This discovery deepens our understanding of HOTAIR's molecular mechanisms across various cancer types and provides a new direction for future studies into HOTAIR's function in other tumor types.

An unexpected finding of this study is that EV‐mediated HOTAIR expression can significantly upregulate PD‐L1 levels, thereby suppressing T‐cell cytotoxicity. This result was beyond our initial expectations and offers a new perspective on the role of LncRNA in immune evasion. PD‐L1, a key molecule in tumor immune evasion, inhibits T cell activity by binding to PD‐1, helping tumor cells evade host immune surveillance. Our finding that HOTAIR upregulates PD‐L1 suggests that HOTAIR may be involved in regulating immune checkpoint pathways. Unlike other studies, research on the interaction between LncRNAs and PD‐L1 remains limited, particularly regarding EV‐mediated LncRNA regulation of PD‐L1. This unexpected result indicates that HOTAIR may enhance immune evasion through multiple pathways, providing a new direction for exploring LncRNA roles in immune checkpoint blockade therapies.

The EVs‐HOTAIR regulatory mechanism revealed in this study offers a novel strategy for targeted therapy in EC with significant clinical application potential. Blocking HOTAIR or its downstream regulatory molecules (such as CDH2 and PD‐L1) could lead to new clinical therapies, improving chemotherapy responses to PTX and reducing immune evasion in EC patients. Based on our findings, we recommend investigating targeted HOTAIR inhibitors in clinical practice to reduce tumor resistance and combining them with PD‐L1 inhibitors to further enhance immune efficacy. Additionally, HOTAIR could be a predictive biomarker to identify EC patients at high risk of PTX resistance and immune evasion, facilitating personalized treatment strategies and improving therapeutic outcomes.

Despite the important advancements of this study, there are some limitations. Although we employed various experimental techniques, such as dual‐luciferase reporter and RNA pull‐down assays, these methods are cell‐based and may not fully represent the complex tumor microenvironment in vivo. Future studies could incorporate in vivo imaging technologies or clinical sample analysis to further verify the role of EVs‐HOTAIR in EC patients.

Based on the findings of this study, several unresolved issues require further investigation. First, the specific secretion mechanism of HOTAIR in EVs remains unclear. Further research into whether different types of EVs exhibit distinct LncRNA loading mechanisms could provide a more comprehensive understanding of EVs' regulatory functions. Additionally, the potential impact of HOTAIR on other miRNAs or signaling pathways should be explored to fully understand its complex role in the EC microenvironment. Future research should prioritize validating these findings in animal models and expanding to other cancer types to explore the broader applicability of EVs‐mediated HOTAIR mechanisms within the tumor microenvironment. These studies lay the theoretical foundation for developing targeted therapies that can be broadly applied across different cancers.

This study reveals that tumor cell‐derived EVs‐LncRNA HOTAIR, that upregulates CDH2 expression by sequestering miR‐375, promotes immune evasion and PTX resistance in EC cells. This finding provides new evidence of the roles of EVs and LncRNAs within the tumor microenvironment. It enhances our understanding of the molecular pathology of EC but also provides new molecular targets for future targeted therapies. The scientific value of this study lies in expanding our understanding of the roles of EVs and LncRNAs in cancer resistance and immune evasion mechanisms, whereas its clinical significance rests in offering potential new therapeutic strategies for EC patients. Future studies should aim to verify these results across different cancer types and explore more precise targeted intervention strategies to facilitate the clinical translation of EVs‐HOTAIR.

## 5 CONCLUSION

This study reveals the key role of tumor‐derived EVs‐LncRNA HOTAIR in EC immune evasion and PTX resistance. HOTAIR upregulates CDH2 expression by sequestering miR‐375, promoting immune evasion and PTX resistance in EC cells. Through a series of experiments, we confirmed the direct interaction between HOTAIR and miR‐375, demonstrating that HOTAIR enhances immune evasion by modulating PD‐L1 expression, thus increasing cancer cell resistance to T cell‐mediated immune attacks. Further investigation showed that targeting CDH2 could partially reverse HOTAIR‐mediated PTX resistance and immune evasion effects.

These findings offer new insights into the molecular mechanisms underlying PTX resistance and immune evasion in EC. Given the critical role of LncRNA HOTAIR in EC progression, targeting HOTAIR or its downstream regulatory axis (such as the miR‐375/CDH2 and PD‐L1 pathways) could present novel therapeutic strategies for EC patients, particularly for those exhibiting PTX resistance. A limitation of this study is that it was primarily based on in vitro experiments, lacking further validation in animal models. Clinical validation in EC patient samples is also essential. Future research should explore the feasibility of HOTAIR‐targeted therapies in clinical settings and assess their safety and efficacy, especially regarding their potential in combination with existing immunotherapy and chemotherapy regimens.

## AUTHOR CONTRIBUTIONS

Tayier Tuersong and Munire Shataer conducted the experiments and performed data analysis. Yan Chen and Xiaoling Li contributed to the experimental design and provided technical support. Gaosi Chen assisted in data interpretation and manuscript revision. Linjie Lei participated in data collection and figure preparation. Ayiguli Younusi and Liangying Ma conceived and supervised the study, secured funding, and critically revised the manuscript. All authors read and approved the final version of the manuscript.

## CONFLICT OF INTEREST STATEMENT

The authors declare no conflicts of interest.

## ETHICS STATEMENT

None.

## Supporting information

Supporting Information S1

## Data Availability

All data can be provided as needed.
