# Correction to “Extracellular vesicle‐LncRNA HOTAIR modulates esophageal cancer chemoresistance and immune microenvironment via miR‐375/CDH2 pathway”

**DOI:** 10.1002/ccs3.70065

**Published:** 2026-02-20

Tuersong, T., Shataer, M., Chen, Y., Chen, G., Li, X., Lei, L., Younusi, A. and Ma, L. (2025), Extracellular vesicle‐LncRNA HOTAIR modulates esophageal cancer chemoresistance and immune microenvironment via miR‐375/CDH2 pathway. *J. Cell Commun. Signal*, 19: e70014. https://doi.org/10.1002/ccs3.70014.

In the originally published article, author Tayier Tuersong's affiliations were given incorrectly. The correct affiliations are shown below. The online version of this article has been corrected.

Incorrect:

Tayier Tuersong^1^, Munire Shataer^2^, Yan Chen^1^, Gaosi Chen^3^, Xiaoling Li^1^, Linjie Lei^1^, Ayiguli Younusi^1^, Liangying Ma^1^

^1^Department of Pharmacy, Xinjiang Key Laboratory of Neurological Diseases, Xinjiang Clinical Research Center for Nervous System Diseases, Second Affiliated Hospital of Xinjiang Medical University, Urumqi, China

^2^Department of Histology and Embryology, Basic Medical College of Xinjiang Medical University, Urumqi, China

^3^Department of Nephrology, Wuhan Children's Hospital, Wuhan Maternal and Child Healthcare Center, Tongji Medical College, Huazhong University of Science & Technology, Wuhan, China

Correct:

Tayier Tuersong^1,2^, Munire Shataer^3^, Yan Chen^1^, Gaosi Chen^4^, Xiaoling Li^1^, Linjie Lei^1^, Ayiguli Younusi^1^, Liangying Ma^1^

^1^State Key Laboratory of Pathogenesis, Prevention and Treatment of High‐Incidence Diseases in Central Asia, Xinjiang Medical University, Urumqi, China

^2^Department of Pharmacy, Xinjiang Key Laboratory of Neurological Diseases, Xinjiang Clinical Research Center for Nervous System Diseases, Second Affiliated Hospital of Xinjiang Medical University, Urumqi, China

^3^Department of Histology and Embryology, Basic Medical College of Xinjiang Medical University, Urumqi, China

^4^Department of Nephrology, Wuhan Children's Hospital, Wuhan Maternal and Child Healthcare Center, Tongji Medical College, Huazhong University of Science & Technology, Wuhan, China

We apologize for this error.

