# Supplementary material for: Extracellular vesicle‐LncRNA HOTAIR modulates esophageal cancer chemoresistance and immune microenvironment via miR‐375/CDH2 pathway
Source: J Cell Commun Signal. 2025 Apr 14;19(2):e70014. doi: 10.1002/ccs3.70014 (PMC11996620; doi:10.1002/ccs3.70014)
Supplement: Supplementary file 1 — Supporting Information S1 [file CCS3-19-e70014-s001.docx]

**
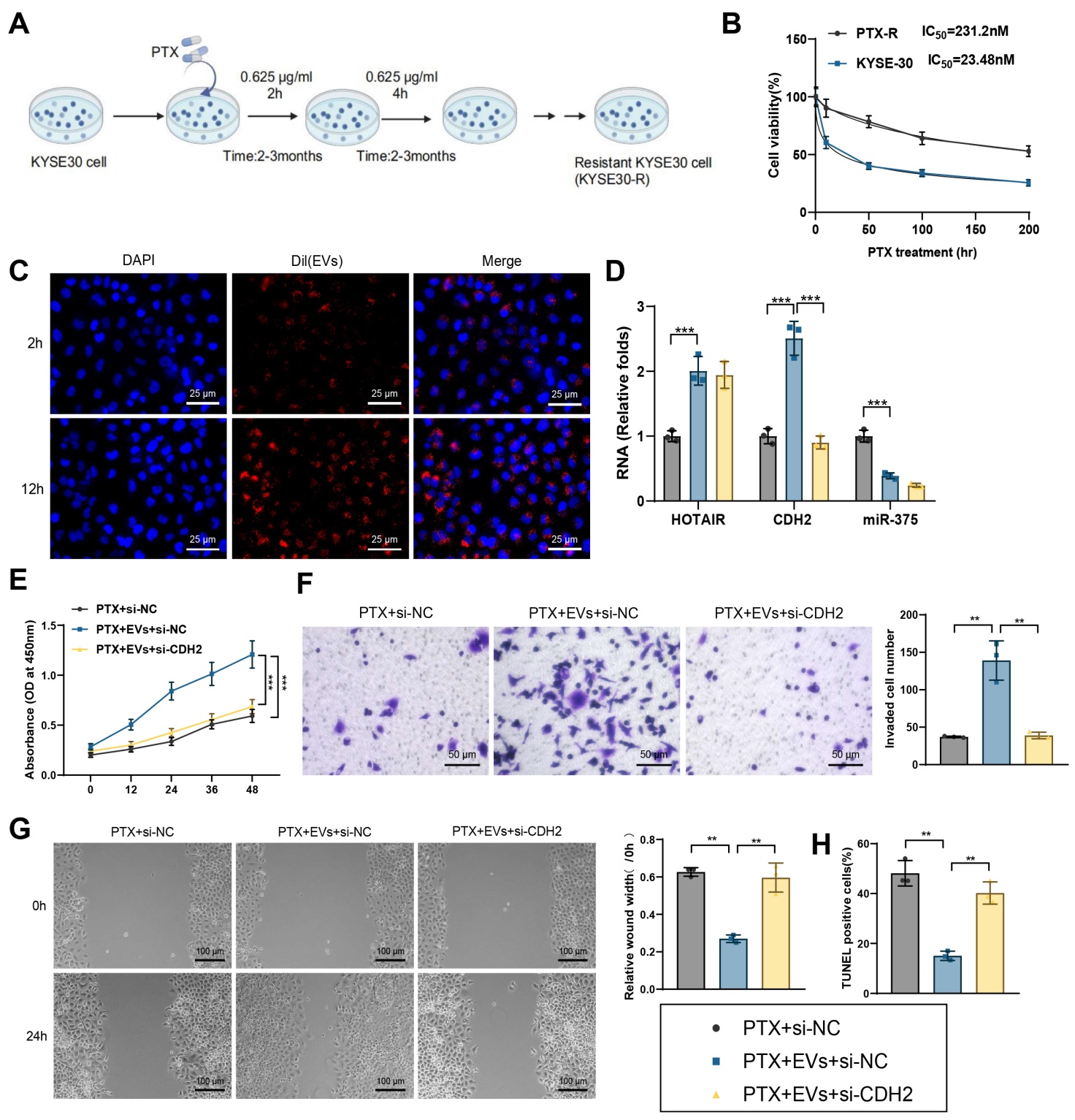
**

**Figure S1. Impact of EV-Mediated miR-375/CDH2 Axis on PTX Resistance in KYSE150 Cells**

Note:(A) Schematic diagram of constructing PTX-resistant KYSE150 cells; (B) CCK-8 assay to assess survival rates of PTX-resistant and PTX-sensitive cell lines after PTX treatment; (C) Fluorescence microscopy to evaluate the uptake efficiency of Dil-labeled EVs by PTX-resistant EC cells; (D) RT-qPCR analysis of HOTAIR, miR-375, and CDH2 mRNA expression levels in resistant cells; (E) CCK-8 assay to assess cell viability across different treatment groups; (F) Transwell migration assay to evaluate the migration ability of resistant cells (scale bar: 50 μm); (G) Scratch assay to assess cell migration in different treatment groups (scale bar: 100 μm); (H) TUNEL staining to analyze apoptosis in drug-resistant cells. Cell experiments were repeated three times. ***p* < 0.01, ****p* < 0.001.

**
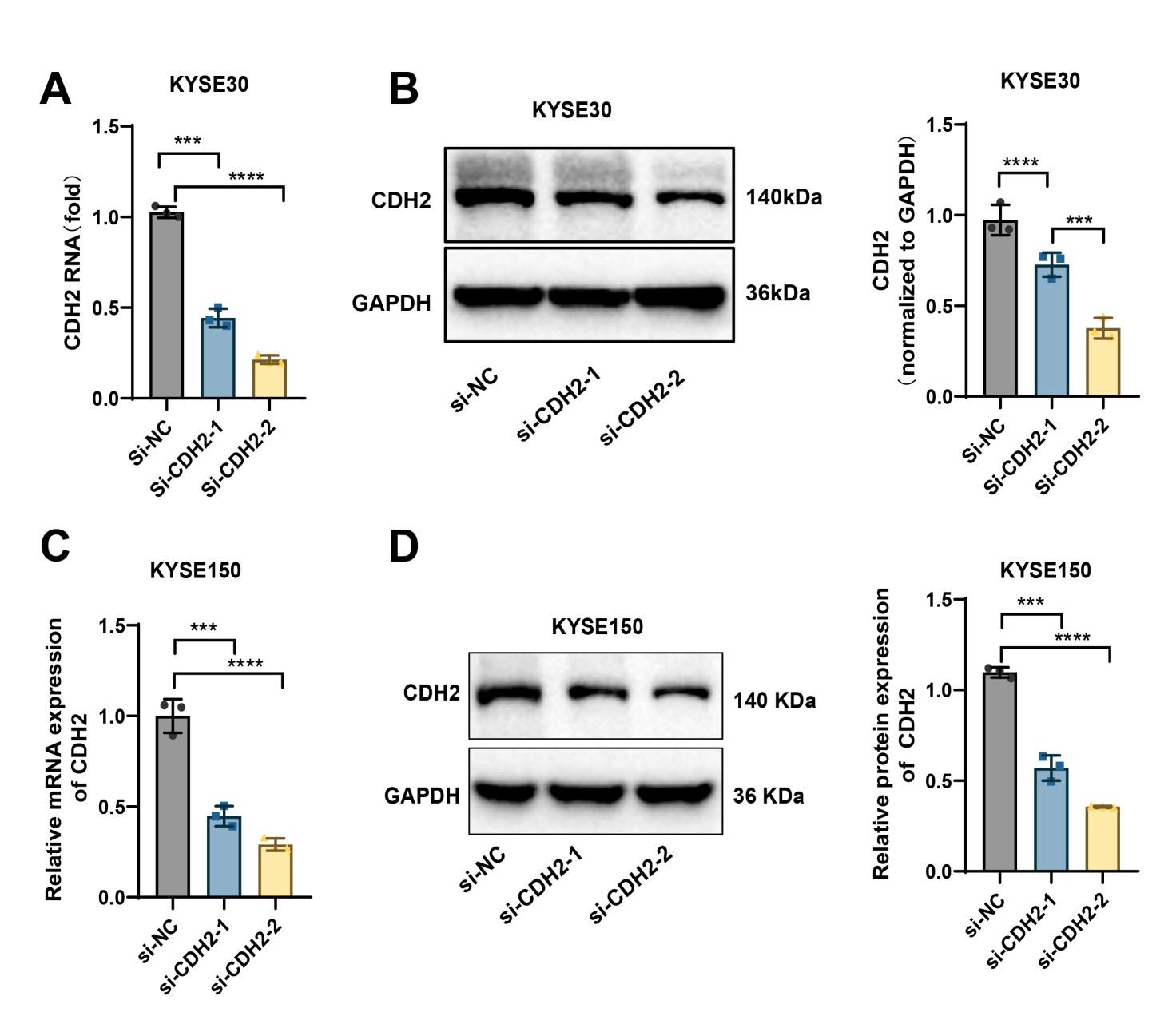
**

**Figure S2. Silencing Efficiency of si-CDH2 in KYSE30 and KYSE150 Cells**

Note:(A-B) RT-qPCR and Western blot analysis of si-CDH2 knockdown efficiency in KYSE30 cells; (C-D) RT-qPCR and Western blot analysis of si-CDH2 knockdown efficiency in KYSE150 cells. ****p* < 0.001.

**
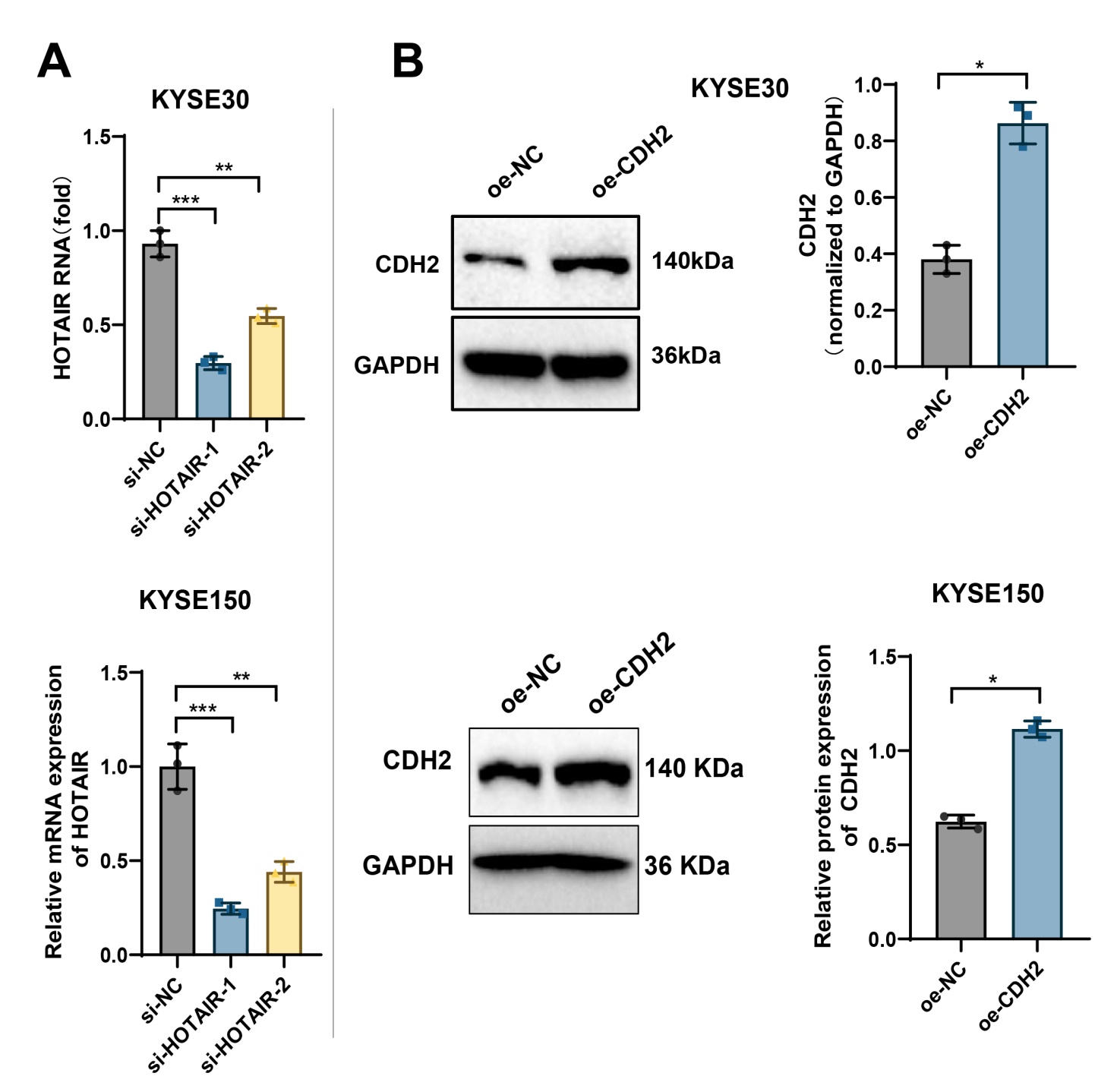
**

**Figure S3. Detection of HOTAIR Silencing and CDH2 Overexpression Efficiency**

Note: (A-B) RT-qPCR analysis of HOTAIR silencing and CDH2 overexpression efficiency. * indicates *p* < 0.051, ** *p* < 0.01, *** *p* < 0.001 compared between groups.

**
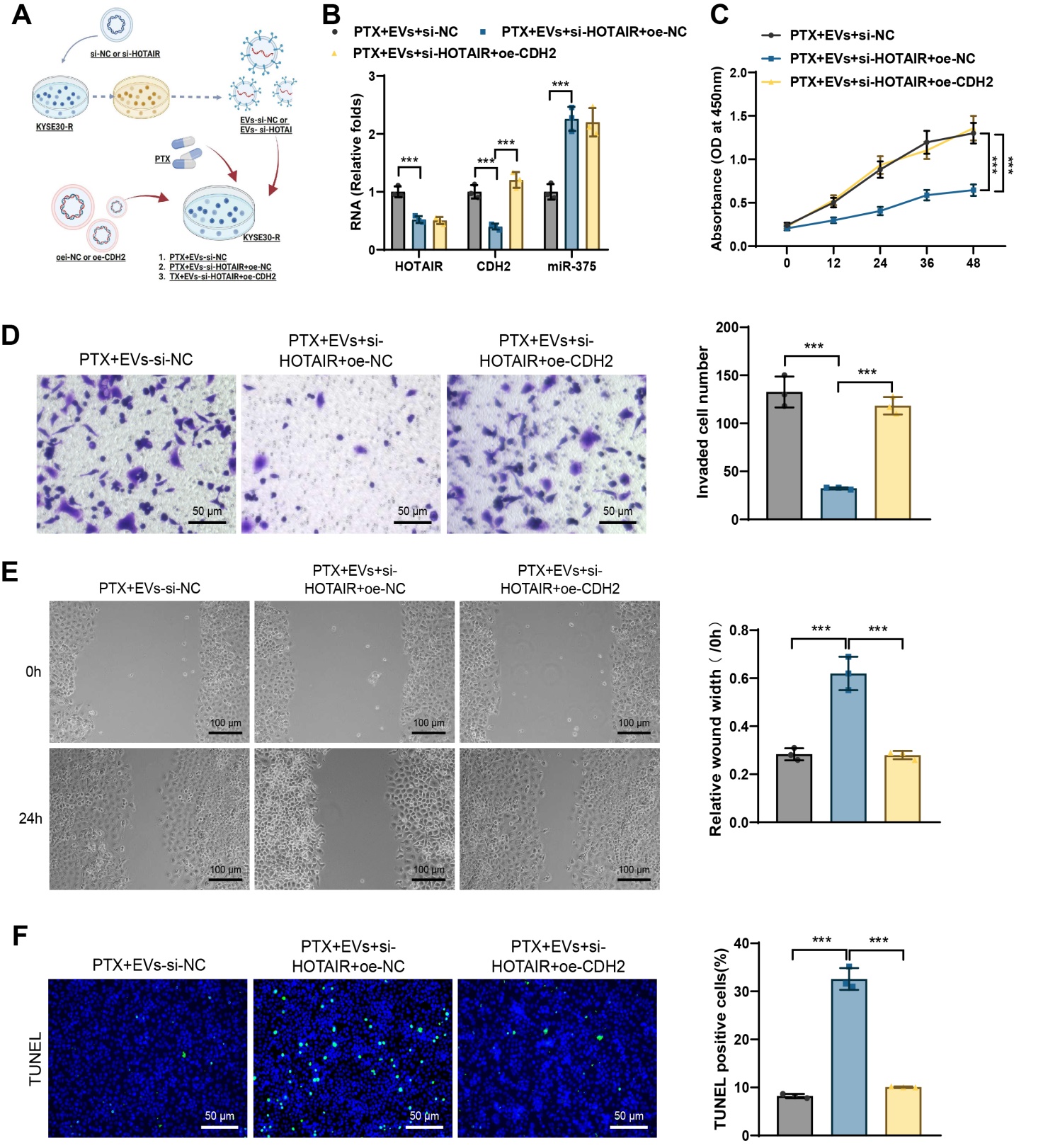
**

**Figure S4. Impact of EV-Delivered LncRNA HOTAIR on PTX Resistance in KYSE150 Cells via the miR-375/CDH2 Axis**

Note:(A) Experimental workflow of EV-mediated LncRNA HOTAIR regulation of the miR-375/CDH2 axis in KYSE150 PTX resistancel; (B) RT-qPCR analysis of HOTAIR, CDH2, and miR-375 expression levels; (C) CCK-8 assay to assess KYSE150 cell viability across different groups; (D) Transwell migration assay to evaluate invasion ability of KYSE150 cells; (E) Scratch assay to assess KYSE150 cell migration; (F) TUNEL staining to evaluate apoptosis rates in KYSE150 cells across different groups. Cell experiments were repeated three times. *** *p* < 0.001 compared between groups.

**
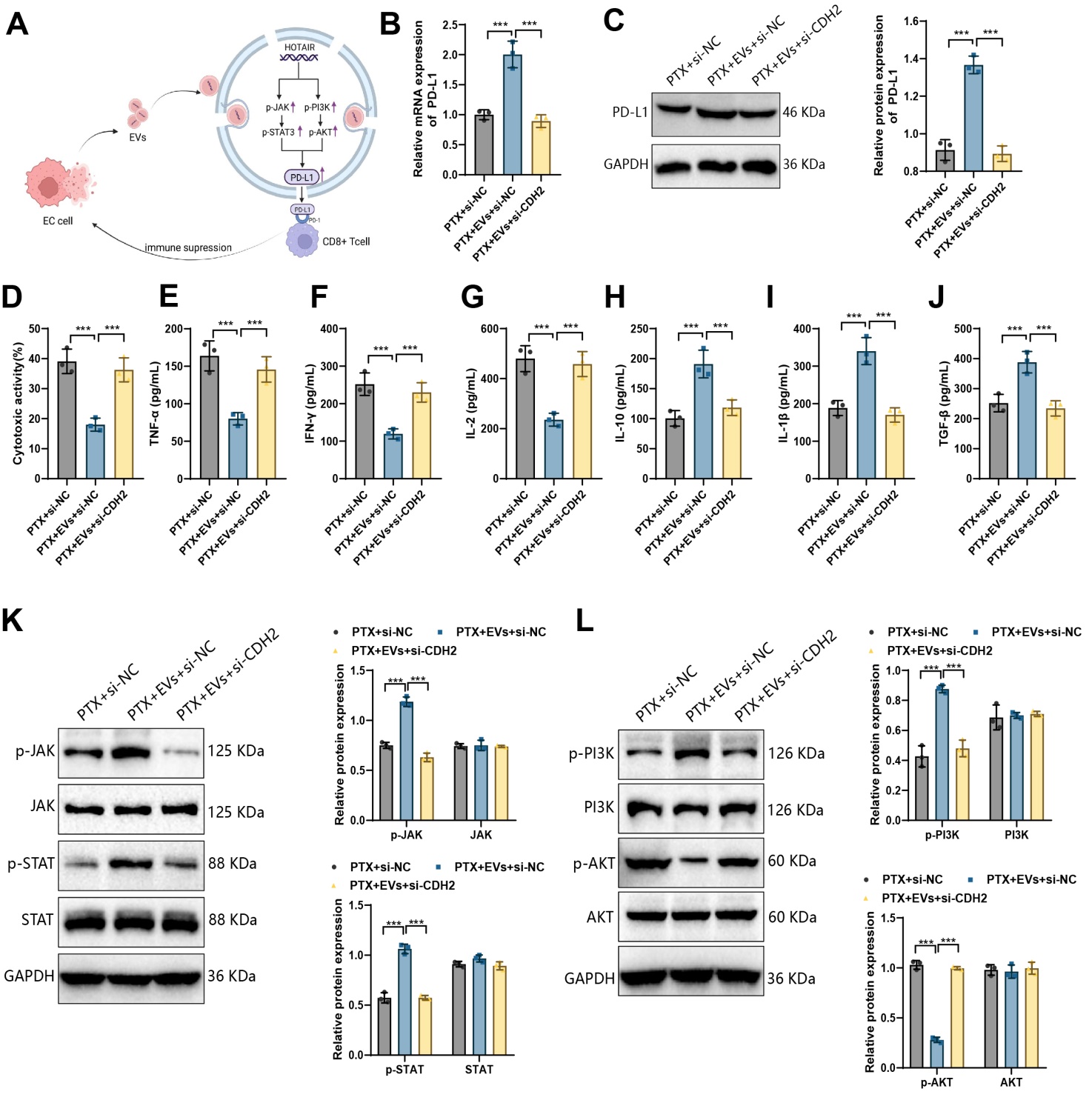
**

**Figure S5. Study of EV-Mediated Immune Evasion Mechanism**

Note:(A) Schematic diagram of the EV-mediated immune evasion mechanism in KYSE150 cells.

(B) RT-qPCR analysis of PD-L1 mRNA expression levels in different KYSE150 treatment groups.

(C) Western blot analysis of PD-L1 protein expression levels in different KYSE150 treatment groups; (D) CCK-8 assay evaluating T-cell cytotoxicity against KYSE150 cells in different treatment groups; (E-J) ELISA measurement of TNF-α, IFN-γ, IL-2, IL-10, IL-1β, and TGF-β levels in co-culture media from different groups; (K-L) Western blot analysis of JAK/STAT and PI3K/AKT signaling pathway activation levels. Cell experiments were repeated three times. ****p* < 0.001.

**
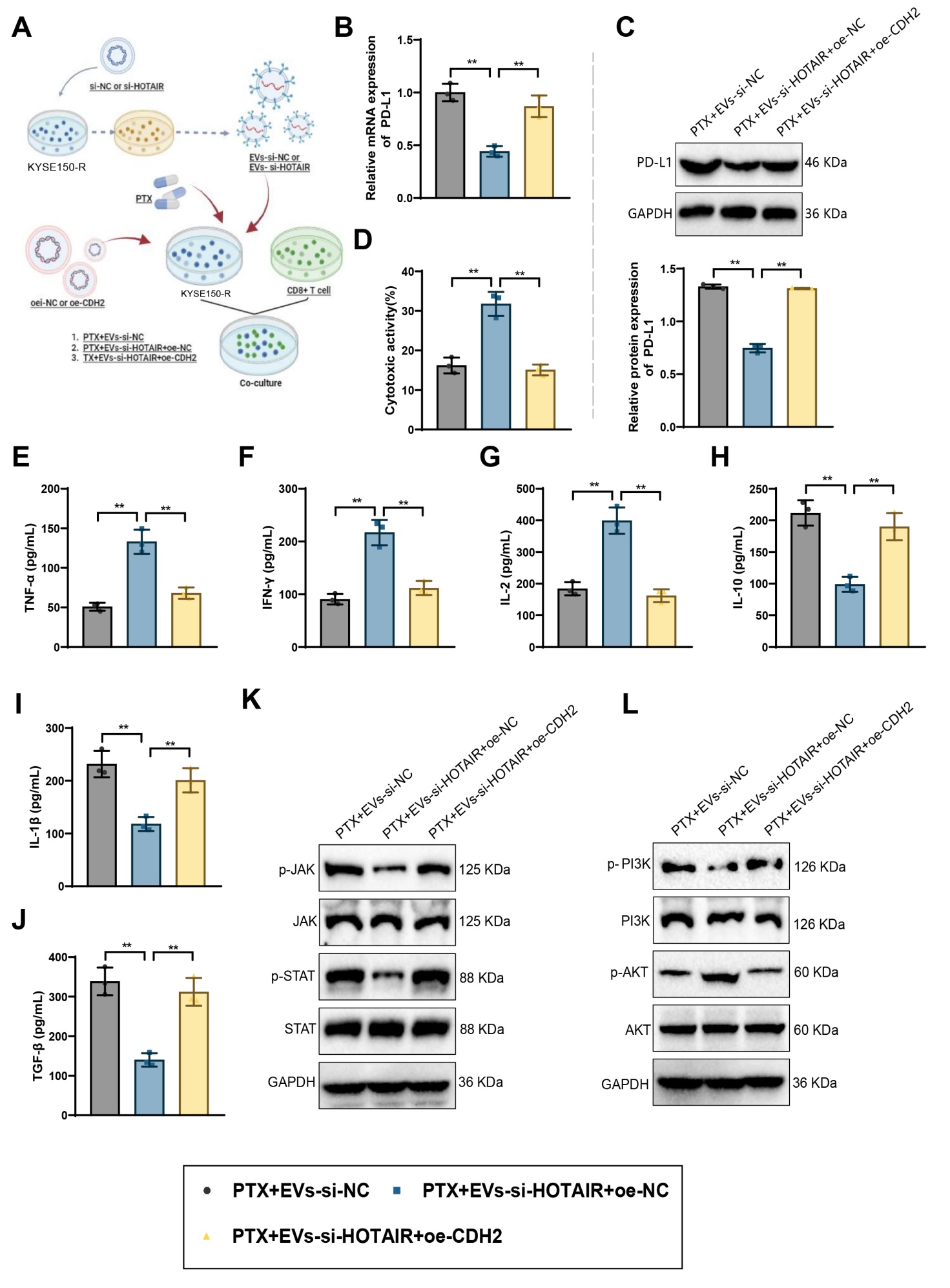
**

**Figure S6. Impact of EV-Delivered LncRNA HOTAIR on Immune Evasion in KYSE150 Cells via the miR-375/CDH2 Axis**

Note:(A) Experimental workflow of EV-mediated LncRNA HOTAIR regulation of the miR-375/CDH2 axis promoting immune evasion; (B) RT-qPCR analysis of PD-L1 mRNA expression levels in different KYSE150 treatment groups; (C) Western blot analysis of PD-L1 protein expression levels in different KYSE150 treatment groups; (D) CCK-8 assay evaluating T-cell cytotoxicity against KYSE150 cells in different treatment groups; (E-J) ELISA measurement of TNF-α, IFN-γ, IL-2, IL-10, IL-1β, and TGF-β levels in co-culture media from different groups; (K-L) Western blot analysis of JAK/STAT and PI3K/AKT signaling pathway activation levels. Cell experiments were repeated three times. ***p* < 0.01. In the figure, Group 1 represents PTX + EVs-si-NC, Group 2 represents PTX + EVs-si-HOTAIR + oe-NC, Group 3 represents PTX + EVs-si-HOTAIR + oe-CDH2.

**Table S1. Cell transfection sequence (human).**

| **Group** | **primer** |
| --- | --- |
| si-NC | 5'-GGGUGAACUCACGUCAGAA-3' |
| si-CDH2-1 | 5'- CUAACAGGGAGUCAUAUGGUGGAGC-3' |
| si-CDH2-2 | 5'- UGCAUAAUGCGAUUUCACCAG-3' |
| si-HOTAIR-1 | 5'- UUCUAAAUCCGUUCCAUUCCA-3' |
| shHOTAIR-2 | 5'- AAAGGAAUCAAUUAAUUAGCG-3' |

**Table S2. RT-qPCR** **primer sequence (mouse).**

| **Gene** | **Primer sequences** |
| --- | --- |
| GAPDH | For GACAGTCAGCCGCATCTTCT |
|  | Rev GCGCCCAATACGACCAAATC |
| U6 | For CTCGCTTCGGCAGCACA |
|  | Rev Universal primer |
| HOTAIR | For GCTCTGGAGCTTGATCCGAA |
|  | Rev GTTCCATTCCACTGCGAAGC |
| miR-375-3p | For TTTGTTCGTTCGGCTCGCGTGA |
|  | Rev Universal primer |
| CDH2 | For GGCGTTATGTGTGTATCTTCACT |
|  | Rev GCAGGCTCACTGCTCTCATA |
| PD-L1 | For TTGCTGAACGCCCCATACAA |
|  | Rev TCCAGATGACTTCGGCCTTG |

Note: For: Forward; Rev: Reverse.

**Table S3. Antibodies used for Western blot analysis.**

| **Antibody** | **Brand** | **Catalog Number** | **Dilution Ratio** |
| --- | --- | --- | --- |
| CDH2(N-cadherin) | Cell Signaling Technology, USA | 13116 | 1:1000 |
| ABCB1 | Thermo Fisher Scientific, USA | MA1-26528 | 1:500 |
| BCL-2 | Cell Signaling Technology, USA | 15071 | 1:1000 |
| LC3 | Cell Signaling Technology, USA | 2775S | 1:1000 |
| PD-L1 | Cell Signaling Technology, USA | 13684 | 1:1000 |
| CD63 | Abcam, UK | ab134045 | 1:1000 |
| CD81 | Abcam, UK | ab79559 | 1:500 |
| TSG101 | Abcam, UK | ab125011 | 1:1000 |
| Calnexin | Abcam, UK | ab22595 | 1:1000 |
| p-JAK2 | Cell Signaling Technology, USA | 3771 | 1:1000 |
| p-PI3K | Cell Signaling Technology, USA | 17366 | 1:1000 |
| p-AKT | Cell Signaling Technology, USA | 4060 | 1:2000 |
| p-STAT3 | Cell Signaling Technology, USA | 9145 | 1:2000 |
| JAK2 | Cell Signaling Technology, USA | 3230 | 1:1000 |
| PI3K | Cell Signaling Technology, USA | 4292 | 1:1000 |
| AKT | Cell Signaling Technology, USA | 9272 | 1:1000 |
| STAT3 | Cell Signaling Technology, USA | 9139 | 1:1000 |
| Anti-rabbit | Cell Signaling Technology, USA | 7074 | 1:2000 |
| Anti-mouse | Cell Signaling Technology, USA | 7076 | 1:2000 |
